# Sleep and Cognitive Dysfunction in Subarachnoid Hemorrhage: A Scoping Review

**DOI:** 10.3390/jcm15031002

**Published:** 2026-01-26

**Authors:** Dayeon Son, Julia K. Veitinger, Revika Singh, Alptug Kaynar, Noreen Hassan, Benedikt Haupt, Fang Yu, Sherry H.-Y. Chou

**Affiliations:** 1 Department of Neurology, Northwestern Feinberg School of Medicine, Chicago, IL 60611, USA; dayeon.son@northwestern.edu (D.S.); julia.veitinger@northwestern.edu (J.K.V.); revika.singh@northwestern.edu (R.S.); noreenhassan2027@u.northwestern.edu (N.H.); 2 Department of Biological Sciences, Columbia University, New York, NY 10027, USA; ahk2181@columbia.edu; 3 Department of Neurological Surgery, Northwestern Feinberg School of Medicine, Chicago, IL 60611, USA; benedikt.haupt@northwestern.edu; 4 Department of Neurology, Westchester Medical Center, Valhalla, NY 10595, USA; fang.yu@wmchealth.org

**Keywords:** SAH, sleep, cognitive dysfunction, patient-centered outcomes, outcomes

## Abstract

Subarachnoid hemorrhage (SAH) is a devastating form of stroke that disproportionately affects younger individuals and often results in long-term disability, even among those who achieve favorable outcomes on traditional clinical scales. This scoping review uses the PRISMA-ScR protocol to evaluate independent studies from 1980 to 2025 and synthesizes current evidence on sleep and cognitive dysfunction in SAH survivors, highlighting their prevalence, impact, and gaps in assessment and management. A total of 2243 publications were screened across three databases (PubMed, EMBASE, and Web of Science), which resulted in 115 studies analyzed for review. Sleep disturbances—including insomnia, hypersomnia, and sleep apnea—are common across all phases of recovery and are closely linked to fatigue, mood disorders, and impaired cognitive function. Cognitive deficits, particularly in memory, executive function, and attention, persist in most survivors and are significant barriers to return to work and reintegration. Despite their clinical relevance, these domains are underrepresented in SAH research and inadequately captured by standard outcome measures such as the modified Rankin Scale. Compared to moderate-to-severe traumatic brain injury (TBI), the SAH literature lacks standardized assessment tools, longitudinal data, and interventional studies. Neuroinflammation may underlie both sleep and cognitive sequelae, offering a potential therapeutic target. This review underscores the need for patient-centered outcome measures, integrated sleep and cognitive assessments, and targeted interventions to improve long-term brain health in SAH survivors.

## 1. Introduction

SAH affects 9–12 per 100,000 person-years, accounts for ~10% of all strokes, and carries 30% mortality [1,2]. Roughly half of patients are <55 years, making SAH a leading cause of stroke-related years of potential life lost before age 65 [1,3,4]. About 85% of cases arise from ruptured intracranial aneurysms [5,6].

Despite declining mortality over the past few decades, SAH survivors continue to experience persistent morbidity across multiple neurological domains beyond focal deficits [6,7]. These include sleep disturbance and fatigue, executive function, memory, and language and mood disorders [5,8,9,10]. Sexual dysfunction, olfactory, and endocrine disturbances represent additional but frequently overlooked sequelae, underscoring the multifaceted burden of SAH survivorship [11,12,13,14]. These disturbances substantially reduce overall quality of life, limit return to work, and lead to significant socioeconomic consequences [12,15,16,17,18,19,20].

The field still relies heavily on outcome assessment tools such as the modified Rankin Scale (mRS) and Glasgow Outcome Scale (GOS) [21,22,23]. Originally designed as global disability scales, these instruments lack the granularity to detect neurocognitive and psychosocial deficits, a limitation most evident with a mRS < 3 where a “favourable outcome” can mask clinically meaningful impairment [24,25]. To date, there is no standardized outcome framework for SAH that captures patient-reported cognitive disturbances within the overall evaluation of functional recovery.

Sleep disturbance is among the most frequent sequelae of SAH, and they strongly correlate with cognitive impairments in a bidirectional fashion. However, they remain absent from standard follow-up assessments after SAH [26,27,28,29,30]. This scoping review synthesizes the current evidence on sleep and cognitive disturbances after SAH, spanning the acute, subacute, and chronic phases, and evaluates the assessment tools available to capture these domains. As a clinical comparator, we reference moderate-to-severe TBI to contextualize the breadth of existing evidence, measurement approaches, and knowledge gaps, and to outline future directions for outcome assessment in SAH. This scoping review aims to address two main objectives through an extensive literature search. (1) To determine the types and prevalence of sleep disorders in SAH survivors across the acute, subacute, and chronic phases of recovery. (2) To determine the types and prevalence of cognitive disorders in SAH survivors across the acute, subacute, and chronic phases of recovery.

## 2. Materials and Methods

### 2.1. Search Strategy

The research question targeted survivors of spontaneous SAH (aneurysmal and non-aneurysmal), sleep dysfunction, and cognitive dysfunction in the context of the acute (0–3 months), subacute (3–12 months), and chronic (>12 months) phases of SAH recovery.

This scoping review was performed using the Preferred Reporting Items for Systematic Reviews (PRISMA-ScR) protocol. We performed searches in PubMed, Embase, and Web of Science using standardized Medical Subheading (MeSh) search terms including “subarachnoid hemorrhage”, “sleep”, “sleep disorder”, and “cognitive dysfunction”. Inclusion criteria for literature used were: original clinical studies (retrospective and prospective) from the year 1980 through July 2025, involving human subjects, adults age ≥ 18 years, study endpoints include sleep, cognition/cognitive domains following SAH, and publications in English. Studies were excluded if they did not contain human subjects, meta-analyses of original clinical studies, reviews, studied sleep as a risk for future SAH development rather than SAH outcome, and if they were gray literature. These searches were compliant with the PRISMA guidelines. The protocol for this scoping review was registered on 19 January 2026 in Open Science Framework (OSF). The link to this registration is https://osf.io/3sha2.

All publications selected for inclusion for this scoping review were systematically classified and analyzed by study authors to extract data on study population, sample size, study design, assessment tools used, and SAH disease phase/time point. Studies were stratified by the time since SAH, defined as acute, subacute, and chronic. Some studies examined different post-SAH time frames that did not conform to the study’s stratification of acute, subacute, and chronic phases. For the studies that crossed multiple strata of time frames, we reported them in their own category.

### 2.2. Data Charting and Synthesis

**Figure 1:**
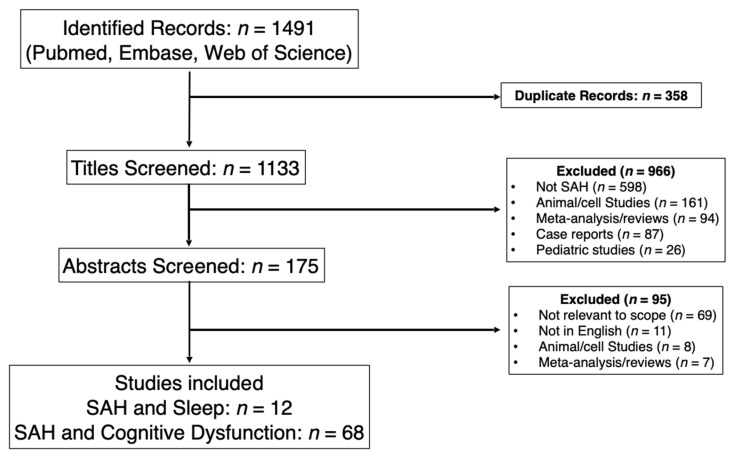
PRISMA Flow Diagram for SAH, Sleep, and Cognitive Dysfunction.

**Figure 2:**
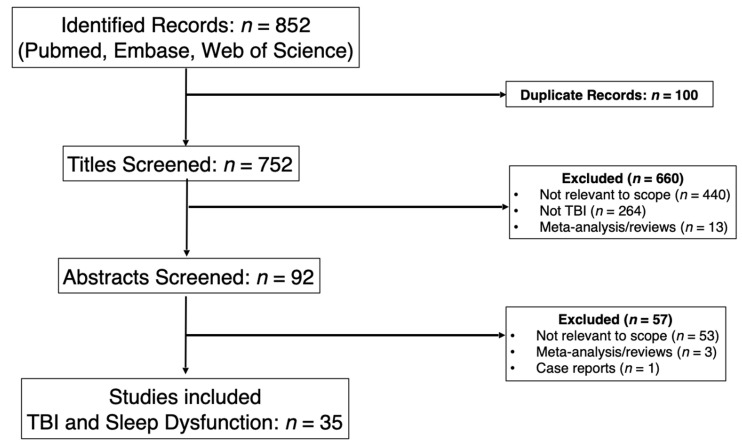
PRISMA Flow Diagram for TBI and Sleep.

Authors RS and NH searched for records from PubMed, EMBASE, and Web of Science. Reviews and literature searches were excluded from the search. The search strategy is charted in Supplemental Table S1. The search resulted in 1491 articles which were subsequently uploaded to Rayyan, a screening platform to assist with the exclusion of duplicates, of which 358 were identified. This left authors DS and JKV with 1133 to screen. Next, the articles were screened for relevance by title. This excluded an additional 966 articles. The most common reason for exclusion was that the article was not about SAH (*n* = 476), or that it was an animal study (*n* = 110). 175 articles moved into the next phase of review. At this point, the abstracts were reviewed. This excluded another 95 articles, with articles not being relevant or for the scope of our work as the most common reason for exclusion (*n* = 65). Please see our PRISMA figures (Figure 1 and Figure 2) for a more detailed breakdown of study exclusionary reasons. For the 80 remaining articles the whole article was read to determine if it fit the review criteria; with 12 SAH and Sleep, and 68 SAH and cognitive dysfunction articles being included.

When reviewing the sleep articles, those that were ultimately included in the review needed to meet the following criteria. They discussed the presence and persistence of disordered sleeping in SAH survivors. Additionally, they needed to use at least one subjective outcome measure to help quantify the degree of disordered sleep. This ultimately yielded 12 studies to include.

When reviewing the cognitive dysfunction articles, the key points that drove the inclusion of an article was primarily if a consistent form of cognitive evaluation was used. This was not restricted to quantitative tools, but also included qualitative tools and structured interviews to glean information about perceived deficits. Additionally, the articles needed to define the time points of evaluation. Ultimately, 68 articles met these criteria and were included in the review.

Given the broad heterogeneity in study designs and assessment tools used in the available studies, applying a uniform risk of bias tool was methodologically challenging and would have offered limited value, as we were not performing a meta-analysis. Thus, we relied on narrative synthesis and did not conduct a risk-of bias assessment. The schematic matrices for SAH Phase x Type of Sleep Disturbance and Cognitive Tool x Phase are displayed in Supplemental Tables S2 and S3.

### 2.3. Comparators

To identify gaps in existing literature, we performed the same PubMed, Embase, and Web of Science searches on TBI using the MeSH terms “traumatic brain injury”, “moderate traumatic brain injury”, “severe traumatic brain injury”, “sleep”, “sleep disorders”, and “cognitive dysfunction”. In this search, we excluded studies that solely focused on mild TBI, studies restricted to the hyperacute phase of TBI, and studies restricted to specialized populations such as geriatrics or veterans, as there were no comparable studies in the SAH literature search. The same timeline for inclusion, 1980 to July 2025 was used. Abstracts were systematically reviewed by study authors based on inclusion/exclusion criteria. With moderate and severe TBI and sleep dysfunction, the search yielded 752 publications. Thirty-five were included for comparison to the 12 SAH and sleep studies.

Ischemic stroke and intracerebral hemorrhage (ICH) patients were not used as comparators because of their distinct pathophysiology, differences in patient demographics and outcomes, and differences in prognostic scales and severity metrics compared to SAH. SAH patients are generally younger and have higher early mortality and neuropsychological sequelae compared to ischemic stroke or spontaneous ICH [31,32]. The WFNS (GCS-based) and/or Hunt and Hess are validated scales to measure initial SAH severity, while the NIH Stroke Scale is used for ischemic stroke and various validated scores such as the ICH score is used for spontaneous ICH [33]. Additionally, mild TBI was not a suitable comparator for patients with spontaneous SAH because SAH patients have a much higher risk for mortality and disability. They typically require hospital and ICU admission, whereas a large proportions of mild TBIs are concussions [33,34,35].

## 3. Results

### 3.1. Sleep Dysfunction in SAH

Sleep disturbances are highly prevalent and contribute to impaired daily functioning, reduced quality of life, mood disorders, increased disease burden, and premature mortality in patients with and without acute brain injuries [36,37]. Poor sleep quality is associated with a generally lower quality of life (e.g., physical functioning, social functioning, mental health), lower psychological well-being (i.e., higher rates of anxiety and depression), and a harder reintegration to normal (including daily tasks, chores, jobs, etc.) [28,38]. Specific to patients with brain injury, sleep plays an important role in maintaining neuroplasticity after stroke, which is crucial for memory consolidation and cognitive function [39].

The six categories of sleep disorders as defined by the third edition of the International Classification of Sleep Disorders (ICSD-3) include insomnia (the inability to initiate or maintain sleep), sleep-related breathing disorders (e.g., obstructive sleep apnea, a total or partial airway collapse), hypersomnolence (excessive daytime sleepiness), circadian rhythm disorders (sleep–wake disturbances due to a shift in the circadian system due to misalignment of sleep/wake times and daily schedules), parasomnia (unusual behaviors during sleep such as sleep-walking, sleep-talking, and night terrors), and sleep-related movement disorders (e.g., Restless Leg Syndrome, the urge to move the legs accompanied by unpleasant sensations in the legs). Despite growing recognition of their clinical importance, many cases remain undiagnosed even in non-brain-injured patients, limiting timely treatment and adversely affecting patient outcomes [40]. Sleep disturbances have been shown to exacerbate neuroinflammation, impair neuroplasticity, and interfere with cognitive rehabilitation [41,42].

#### 3.1.1. Assessment of Sleep in SAH and Severe Brain Injuries

Assessment of sleep quality and sleep dysfunction in stroke and brain-injured patients is more complex, as their injury may confound assessments of sleep function both in qualitative and quantitative methods. Currently, there are no sleep tools validated for the SAH population. There is no commonly used or accepted tool to assess sleep dysfunctions in this population, rather there is a diverse and heterogenous collection of tools that are used. Despite some overlapping domains, data from studies using different sleep assessment tools cannot be integrated or compared with one another, limiting our general knowledge about sleep dysfunction in SAH survivors. This is a gap in the field of sleep disorders. Currently, there is no longitudinal data on sleep dysfunction progression or recovery over time in SAH survivors. Sleep disorders may evolve, resolve, and new ones may arise in the course of a long-term SAH survivor.

Many qualitative questionnaires simply ask whether the patient is experiencing disturbances to their sleep (e.g., “Did you feel tired?” in the SF-36). Fewer tools go deeper to assess and quantify the severity of sleep dysfunction or examine the different aspects of sleep dysfunction. Of the tools reported in this review, some have been specifically validated for use in the stroke patient population, such as the Pittsburgh Sleep Quality Index (PSQI), the Sleep Condition Indicator (SCI), the Richards–Campbell Sleep Questionnaire (RCSQ), the STOP-BANG questionnaire for post-stroke OSA, and the Epworth Sleepiness Scale (ESS). Polysomnography (PSG) remains the gold standard for diagnosing sleep disorders, including in brain-injured patients [43]. In comparison to the general stroke population, there is a notable paucity in the data on sleep assessments in the SAH population.

#### 3.1.2. Sleep Dysfunction Across Different Phases of SAH Recovery

A total of 12 observational cohort studies on sleep dysfunction post-SAH are included. Two studies examine the acute phase [28,44], two examine the subacute [45,46], and 8 studies report on sleep dysfunction experienced in the chronic phase [28,44,47,48,49,50,51,52], including studies that report on 20+ year survivors [48] (Table 1). Of the 12 studies, only 6 reported on specific types of sleep disorders, while the remaining 6 studies used general screening questionnaires to report the presence or absence of sleep disturbances.

In the 6 studies that examined specific types of sleep dysfunction, all found that survivors reported hypersomnia or excessive daytime sleepiness. Five out of 6 (83.3%) reported the presence of insomnia and poor quality of sleep (25–46% of survivors) (Table 1, Lines 12 and 14). Poor sleep quality includes the following specific domains: longer sleep latency (how long it takes to fall asleep), decreased amounts of sleep, lower habitual efficiency (the percentage of time an individual spends asleep compared to the total time in bed), functional disorder during daytime (e.g., excessive sleepiness, fatigue, pain, cognitive dysfunction), and increased sleeping pill dosage. Five out of 6 (83.3%), found that the study cohort also reported fatigue associated with sleep dysfunction (42.6–55% of survivors) (Table 1, Lines 14 and 15). Three out of 6 studies (50%) reported repeated awakening throughout the night (26–37% of survivors), two (33.3%) found SAH patients report too little or too much sleep (33–41% of survivors) (Table 1, Lines 3, 4 and 15) [55]. Of all the studies included, two (33.3%) studies specifically examined sleep apnea symptoms (45.1–95% of survivors) (Table 1, Lines 9 and 14) [28,44,52,55].

##### Key Learnings from the Past 10 Years of Research

Eight of the 12 (66.67%) studies were published in the last 10 years. In the acute and subacute phase 8–73% SAH survivors report poor quality of sleep, with 26–28% of patients experiencing daytime sleepiness and 8–41% experiencing too little or too much sleep (Table 1, Lines 3–5, 7 and 8) [53]. At 1 year following SAH, the prevalence of sleep dysfunction remains high, at 25–51.2% (Table 1, Lines 14 and 16) [28,45,53]. Four of the eight (50%) were classified as chronic highlighting that 18–49% of patients reported a wide range of sleep disturbances from difficulty initiating and maintaining sleep (Table 1, Lines 13 and 15), insomnia (Table 1, Line 14), daytime sleepiness (Table 1, Line 15), sleep apnea (Table 1, Line 14), and fatigue (Table 1, Lines 14 and 15). Even in SAH patients who were classified as having achieved a favorable outcome at discharge (mRS 0–2), 51.2% report having sleep problems beyond 1 year following SAH (Table 1, Line 16) [47].

#### 3.1.3. Sleep Dysfunction in SAH Compared with Moderate to Severe TBI

We selected moderate to severe TBI rather than ischemic stroke or primary ICH populations as a clinical comparison population for the following reasons: (1) Age distribution and underlying co-morbidities profile in moderate to severe TBI are more similar to that of SAH than the ischemic stroke or primary ICH population [56], (2) Moderate to severe TBI patients can sustain both focal and global brain injury, which is more similar to SAH than ischemic stroke or ICH; (3) The recovery phenotypes in TBI are more similar to SAH than ischemic stroke or ICH.

There are more studies on sleep dysfunction following moderate to severe TBI as compared to SAH, utilizing specific sleep study tools and more quantitative data compared with SAH. Supplemental Table S4 summarizes the sleep tools that have been used in clinical studies to date in SAH vs. TBI populations.

Similarly to SAH, common disorders in the comparison TBI population include insomnia and hypersomnia [57,58]. TBI studies tend to be more focused on specific sleep disorders and use the gold-standard polysomnography study along with qualitative tools. In TBI patients, sleep disturbances and daytime sleepiness are predictors of poor quality of life independent of cognitive impairments, especially in the domains of pain, general health, vitality (having energy), and emotional role functioning (how well one can express and regulate their emotions to navigate their social environment). Sleep apnea is associated with additional sleep disorders such as excessive daytime sleepiness and fatigue that have resulted in prolonged impairments in cognition, level of consciousness, and work status. It is also associated with loss of judgment, irritability, and depression in TBI patients; however this data is lacking in current SAH survivor populations [59,60].

More studies examined the incidence and impact of sleep related breathing disorders, more specifically obstructive OSA in the TBI population compared to SAH. OSA is one of the most common sleep disorders during TBI recovery [61]. In comparison, there is a paucity in SAH studies that specifically focus on OSA, and only one study reports on OSA incidence [28]. Another important difference is that TBI, there are interventional studies that examine potential treatment for sleep dysfunction, whereas all SAH studies on sleep dysfunction are descriptive. To date, there is no data on potential interventions for sleep dysfunction in the post-SAH population. All of these factors illustrate a significant knowledge gap in sleep dysfunction in SAH survivors.

The “gold standard” of measuring sleep disturbances is PSG and actigraphy [62]. They provide the most accurate and detailed data based on the patient’s movements and physiology through quantitative data. Only two SAH studies utilized these gold standard, quantitative methodologies, while 14 out of 35 (40%) TBI studies did. This further highlights the knowledge gap and data paucity in SAH and sleep dysfunction. Supplemental Table S4 illustrates the sleep tools that were used in both SAH and TBI studies, Although the PSG and actigraphy are the quantitative and gold standard diagnostics for sleep dysfunction, they do not capture the patients’ experiences. Further studies in SAH would benefit from using both quantitative and qualitative measures to fully capture the extent and impact of sleep dysfunction in SAH.

#### 3.1.4. SAH, Sleep, and Fatigue

Disturbances to sleep–wake cycles play a direct role in fatigue. Both fatigue and sleep disorders are common complaints from SAH survivors and are highly intertwined, along with cognitive recovery. Fatigue can be defined anywhere from “general tiredness” to “weariness not ameliorated by rest [16]. “Not only does it affect daily life, but it can also have negative impacts on a survivor’s mood, memory, and executive functioning”. SAH outcomes studies that examined fatigue as a symptom consistently found that the majority (over 50%) of participants were experiencing some extent of fatigue in their lives. Additionally, persistent fatigue is highly associated with chronic reduction in the quality of life of SAH survivors [16].

Fatigue as a persistent symptom likely also impacts an individual’s overall cognitive health [48,63], Even SAH patients classified as having achieved a favorable outcome report significant subsequent fatigue. SAH survivors with severe fatigue symptoms have less favorable long-term outcomes, including impairments to activities of daily living, depression, and anxiety [64]. Standardized SAH outcome measures used in clinical trials, such as the GOSE or the mRS focus more on physical disability but are less able to capture the “invisible” long-term symptoms such as fatigue, cognitive dysfunction, and sleep disturbances, which have profound implications in the individual’s overall physical and brain health [63,64].

### 3.2. Cognitive Dysfunction in SAH 

Even among SAH survivors who achieve favorable scores on traditional outcome scales (e.g., mRS 0–2), cognitive deficits are highly prevalent and significantly impair long-term recovery. Studies show that up to 71% (Table 2, Row 37) of SAH patients exhibit deficits in at least one cognitive domain—most commonly memory (51%) (Table 2, Row 37), executive function (36%) (Table 2, Row 37), and attention (21%) (Table 2, Row 37)—even one-year post-hemorrhage [65]. These impairments directly affect the ability to resume professional roles and daily responsibilities. For instance, only one third of previously employed SAH survivors fully return to their original jobs [66,67]. Cognitive dysfunction—not physical disability—is often the limiting factor, with executive impairments and slowed processing speed emerging as key predictors of incomplete return to work. ICU clinicians are uniquely positioned to initiate early screening and referral for neuropsychological evaluation, which can guide targeted rehabilitation and improve long-term outcomes. Recognizing and addressing cognitive sequelae is essential not only for clinical completeness but also for restoring patients’ autonomy, productivity, and quality of life.

#### 3.2.1. Cognitive Dysfunction and Sleep

Sleep is a critical determinant of cognitive function, playing a central role in memory consolidation, executive functioning, and emotional regulation. In the context of SAH, where cognitive impairments are prevalent even among patients with favorable functional outcomes, the role of sleep in recovery is increasingly recognized but remains underexplored [44]. Emerging data suggest that around 71% (Table 2, Row 37) of SAH survivors experience cognitive deficits in at least one domain, with memory and executive function being the most commonly affected. Even those with a favorable outcome (mRS 0–2), a majority of patients (60%) (Not in table because it is a review article) experience cognitive deficits, demonstrating that cognitive deficits persist and are present in the vast majority of patients [65,131].

The etiology of such long-term cognitive dysfunction in SAH is not well understood. Clinical evidence on the association between initial clinical SAH severity and long-term cognitive outcomes is inconsistent [132].

The overall contribution of post-SAH cognitive function and recovery is likely multifactorial and complex. Neuroplasticity is a key component in recovery after an acute brain injury. Emerging evidence in TBI shows that sleep disturbances negatively impact synaptic and neuronal plasticity, suggesting that sleep disturbances may be part of the underlying pathophysiologic mechanisms of cognitive dysfunction and reduced cognitive recovery following severe brain injury. Given the extent of persistent sleep dysfunction observed in emerging studies in SAH survivors, it is plausible that additional mediating factors such as sleep dysfunction, may mediate some of this disconnect between functional outcomes and cognitive function [15]. If so, sleep dysfunction may be a modifiable factor in post-SAH care.

#### 3.2.2. Cognitive Dysfunction Following SAH

The findings summarized in Table 2 underscore the widespread and enduring nature of cognitive dysfunction following SAH, particularly aneurysmal SAH (aSAH). Cognitive impairment is reported across all phases of recovery—from acute to chronic—and affects a wide range of domains including memory, executive function, attention, psychomotor speed, and verbal fluency.

For example, Wang et al. (2024) found that 78.4% (Table 2, Row 3) of aSAH patients had cognitive impairment at two months post-bleed, while Geraghty et al. (2020) reported that 56.2% (Table 2, Row 4) of patients had mild cognitive impairment (MoCA < 22) prior to discharge [69,70]. Even among patients with good functional outcomes (mRS 0–2), nearly half still exhibited cognitive deficits. Long-term studies also revealed persistent impairments: Persson et al. (2019) found that 61% (Table 2, Row 50) of patients had cognitive impairment seven years post-SAH, and Sonesson et al. (1987) reported that 89% (Table 2, Row 21) of patients had memory impairments up to eight years after the event [55,111].

A major takeaway from the table is the lack of standardization in cognitive assessment across studies. Researchers employed a wide array of tools—from brief screening instruments like the MMSE and MoCA to more comprehensive domain-specific batteries such as the TMT, Benton Test, and VTS. Despite this variability, certain domains emerged as the most affected: memory, executive function, attention, and psychomotor speed. Rass et al. (2024) found that 71% (Table 2, Row 37) of patients had deficits in at least one domain, with memory (51%) (Table 2, Row 37) and executive function (36%) (Table 2, Row 37) being the most common [65]. Ørbo et al. (2008) reported that 93% (Table 2, Row 41) of patients had impairments in memory, executive function, or information processing one year post-surgery [103]. Notably, cognitive dysfunction was observed across all H&H grades, with low-grade patients (H&H 1–3) not being spared. Rowland et al. (2021) found that 44% (Table 2, Row 5) of low-grade aSAH patients were cognitively impaired at three months, while Sonesson et al. (1987) reported severe dysfunction in 18% (Table 2, Row 47) of patients regardless of early or late surgical intervention [55,71]. These findings emphasize that cognitive sequelae are not limited to high-grade SAH and that all survivors.

##### Key Learnings from the Past 10 Years of Research

Over the past decade, research has increasingly highlighted that cognitive dysfunction in SAH is both common and persistent, affecting patients across all stages of recovery. In the acute phase, studies such as Wang et al. (2024), Geraghty et al. (2020), Rowland et al. (2021), and Rautalin et al. (2020) show that between 40–78% (Table 2, Rows 3–5) of patients experience impairments in attention, memory, executive function, and processing speed, even when functional outcomes appear favorable [69,70,71,75]. In the subacute phase, findings from Kälin et al. (2025), Khosdelazad et al. (2024), Jorna et al. (2024), Shen et al. (2018), and Ma et al. (2021) demonstrate that deficits remain in 30–50% (Table 2, Row 18, 20, 25, 29, 33) of survivors, with executive dysfunction and attention problems particularly resistant to recovery [82,84,89,93,97]. Psychiatric comorbidities such as depression, anxiety, PTSD, and fatigue (Table 2, Row 21, 22, 34) further compound the cognitive burden [85,86,98]. In the chronic phase studies including Rass et al. (2024, 2020), Eagles et al. (2019), Gaastra et al. (2022), and Wenneberg et al. (2022) reveal that 30–70% (Table 2, Row 37, 39, 59, 64, 65) of patients remain impaired years later, most often in memory and executive domains [9,65,101,120,124]. These studies underscore the persistence of cognitive dysfunction throughout SAH recovery.

#### 3.2.3. Cognitive Dysfunction Assessments Following SAH

Given the prevalence and impact of cognitive dysfunction on long-term outcomes following SAH, accurate assessment is essential for guiding clinical decision-making, tailoring rehabilitation strategies, and evaluating treatment efficacy [133]. Like sleep, the tools that have been applied to post-SAH cognitive dysfunction assessments are numerous, heterogeneous, and differ significantly between studies. Supplemental Table S4 summarizes the numerous tools that assess cognitive dysfunction in SAH survivors; over 80 tools are cited highlighting the lack of standardization. The MoCA and MMSE are the most common tools to assess global cognition in SAH survivors [65,69,70,105].

##### Montreal Cognitive Assessment (MoCA)

The MoCA is a 30-point screening tool that evaluates a broad range of cognitive domains, including visuospatial/executive function, naming, attention, memory, language, abstraction, and orientation. Clinically, MoCA scores correlate with functional outcomes and the severity of DCI, a major contributor to post-SAH cognitive decline. A change of 2 points on the MoCA has been identified as the minimum important difference, providing a benchmark for interpreting meaningful clinical change. This makes MoCA a potentially valuable tool for monitoring recovery trajectories, assessing treatment efficacy in clinical trials, identifying patients at risk for poor outcomes, and guiding neurorehabilitation planning. Its multidomain coverage and sensitivity make it useful in both acute and long-term follow-up settings [133].

While the standard MoCA can test all cognitive domains, it is more time-consuming and must be administered in person. Modified versions of the MoCA exist to accommodate various clinical situations, such variants are outlined in Supplemental Table S6. Please see the supplement for additional discussion on MoCA variants.

##### Mini-Mental Status Examination (MMSE)

The MMSE, while widely used, is less sensitive to executive dysfunction and subtle cognitive deficits often seen in SAH [133]. It focuses more on orientation, attention, memory, and language, and may miss impairments in domains like mental flexibility and visuospatial construction.

Despite its limitations, MMSE remains useful for baseline cognitive screening, tracking gross changes in cognition, and complementing other assessments in a multimodal battery. However, clinicians should be cautious in relying solely on MMSE for decision-making in SAH patients, especially when executive function is a concern [133]. The MoCA has demonstrated superior sensitivity compared to the MMSE in detecting mild cognitive impairment and executive dysfunction, which are common in SAH patients [134].

##### Beyond the MoCA and the MMSE

General cognitive screening tools like MoCA and MMSE are useful but may overlook deficits in specific domains commonly affected by SAH. SAH survivors experience severe deficits in many domains of cognition, limiting their ability to return to work and integrate back into their lives [63,135,136]. When looking within the same population of SAH survivors, only a subset maybe classified as globally impaired per the MoCA or MMSE a significantly larger proportion of individuals may be impaired specific domains such as memory, task switching, and executive functioning abilities [63,136]. Leveraging domain-specific tools in these domains may allow clinicians to help their patients recover more completely. However, there are no standardized recommended tools for assessing and classifying deficits in these domains.

#### 3.2.4. Cognitive Dysfunction, Return to Work, and Reintegration

Return to work after SAH remains a significant challenge, even for patients without physical disability or achieve “favorable” scores on global outcome measures such as the mRS or the GOSE. Many survivors experience domain-specific cognitive impairments—particularly in executive functioning, memory, and processing speed—not captured by general screening tools like the MoCA or MMSE [66,85]. These deficits directly impact the ability to manage complex tasks, adapt to dynamic environments, and sustain attention, all of which are essential for successful vocational reintegration [137]. Domain-specific cognitive testing is therefore critical for identifying barriers to recovery and guiding individualized rehabilitation strategies.

As summarized in Supplemental Table S5, when trying to identify cognitive domain-specific impairments that are impeding an individual’s ability to return to work (RTW) or reintegrate into normal life (RNL), there is no standardized or widely accepted tool. Understanding the extent and impact of impairment in each domain could help drive focused cognitive rehabilitation and allow more patients to have a true “favorable outcome”.

Executive Function is critical in an individual’s ability to RTW and RNL [138]. There are many tools used to assess an individual’s executive function ability, as it is critical for return to work and reintegration into normal life. Some of the most common include the TMT-B, the Stroop Word-Color Test, and the DEX, all of which were able to detect key differences associated with an individual’s ability to RTW [136].

Memory is another domain that is disproportionately impaired after SAH. The 15WT is one of the most common tools used to assess an individual’s memory objectively [136]. While memory impairments are widespread and while they were not associated with a difference in one’s ability to return to work [12]. Qualitative research has demonstrated that memory impairments greatly reduce the survivors’ long-term quality of life [67,139].

For processing speed, the TMT-A is commonly used, and worse scores have been correlated with an incomplete ability to RTW. Additionally, the Stroop Word Test was superior at detecting impairment; however, it did not translate into an impact on an individual’s ability to RTW [136].

Domain-specific testing offers clinicians a deeper personalized way to identify and address impairments limiting their patients’ quality of life and ability to RNL and RTW. The lack of standardization in tools used highlights a clear gap and a lack of understanding of the extent of the problem.

### 3.3. Neuroinflammation and Cognitive Recovery in SAH

Neuroinflammation is a key contributor to both early and delayed cerebral injury, as well as the long-term outcomes of patients who survive SAH, including cognitive decline, mood disorders, and impaired quality of life [140]. For these reasons, studies have proposed neuroinflammation as a potential therapeutic target [33]. While genetic and other demographic factors may also influence outcomes, persistent neuroinflammation is associated with long-term cognitive deterioration and increased risk of dementia [141]. Clinical studies have demonstrated that elevated inflammatory markers in CSF correlate with worse neuropsychological outcomes [73]. In animal models, experimental therapies targeting neuroinflammation, such as microglial modulations through pathways including AMPK/TBK1/NF-kB and TLR4/NF-kB, Th17/Treg cell homeostasis, and astrocyte modulation, have reduced neuroinflammation after SAH and are associated with improved outcomes, including better cognitive recovery and memory performance [142,143,144,145,146].

Neuroinflammation has also been implicated in disrupted sleep architecture and circadian rhythms [147]. Specifically, inflammatory cytokines, such as IL-1β, TNF-α and IL-6, can impair sleep structure by decreasing slow-wave sleep and increasing nocturnal awakenings, as well as by interfering with the synthesis of melatonin and key neurotransmitters such as serotonin [148,149,150,151,152]. Another proposed hypothesis includes microglial activation altering signaling in brain regions that regulate sleep cycles [153]. A recent prospective imaging study suggests that although neuroinflammation is prominent in the early and delayed phases of SAH-associated cerebral injury, long-term cognitive impairment might be more directly linked to microstructural brain damage rather than persistent neuroinflammation [154]. Thus, post-SAH chronic deficits such as sleep disturbances and cognitive impairment may represent a combination of early neuroinflammation and chronic neuronal and microstructural brain injury. Whether early interventions targeting neuroinflammation in the acute and subacute phases, which likely contribute to sleep and cognitive impairment, can improve long-term outcomes remains unknown.

Human and translational data suggest elevated biomarkers of inflammation are associated with worse long-term clinical outcomes, though specific causal pathophysiologic processes are insufficiently understood [155,156]. Therapeutics targeting neuroinflammatory pathways have demonstrated success in improving SAH outcomes in animal models, these findings have yet to be translated into humans [142,143,144,145,146]. Emerging data now suggest a growing association between neuroinflammation and sleep dysfunction and with cognitive dysfunctions in various populations, there is little to no data in SAH [148,149,150,151,152].

The translational research road map in understanding the underlying pathophysiology of sleep and cognitive dysfunction must begin by identifying these critical gaps. The first step is to better measure and characterize the magnitude and phenotypes of sleep and cognitive dysfunction at different phases of SAH. To this end, the field will need to identify and develop standardized, validated tools to assess these key outcome domains in future, larger SAH cohorts at different key time points of SAH recovery. Future SAH clinical trials need to include these key outcome domains as important endpoints in addition to mortality and global outcomes measures such as the GOS-e. Once sleep and cognitive function outcomes can be consistently measured using standardized, validated tools in SAH, application of precision medicine approach and future biomarkers studies may identify key endotypes and/or potential underlying mechanisms that can be targeted therapeutically.

## 4. Discussion

Despite advances in acute management and neurocritical care, survivors of SAH often experience persistent cognitive, emotional, and functional challenges that are not fully captured by traditional clinical outcome measures. Global outcome scales such as the mRS and the GOSE have long been considered the gold standard for powering SAH therapeutic trials. While these measures are well-validated in patients with acute brain injuries, they are insensitive to specific impairments that significantly affect patient experience—such as cognitive deficits, fatigue, and difficulties with social reintegration. These domains often matter most to patients.

Moreover, these scales contribute to a “ceiling effect” in SAH outcome data, failing to detect persistent disabilities in patients classified as having achieved a “favorable outcome.” Even among those deemed to have recovered well, many report ongoing challenges in daily life that prevent them from returning to work or resuming pre-hemorrhage responsibilities. Notably, such impairments are present even in patients with full functional recovery, underscoring the limitations of conventional outcome measures. PROMs, especially those incorporating qualitative methodologies, are essential to bridge this gap. These tools prioritize patients’ perspectives and lived experiences, offering a more holistic understanding of recovery. Qualitative approaches—such as semi-structured interviews, focus groups, and patient narratives—allow researchers and clinicians to explore domains often overlooked in quantitative assessments, including fatigue, emotional distress, identity changes, and social reintegration.

In this scoping review, we carefully examined recent clinical studies on sleep and cognition in SAH, incorporating both global and patient-centered outcome measures. Emerging data reveal a strong correlation between sleep disorders, quality of life, psychological well-being, and social reintegration in SAH survivors, highlighting the significant and underrecognized burden of sleep and cognitive dysfunction. Findings from this review expose critical gaps in our understanding and management of these long-term issues—domains that represent major sources of invisible disability and morbidity.

Sleep disturbances are highly prevalent throughout all phases of SAH recovery. These disturbances span multiple domains—including insomnia, hypersomnia, circadian rhythm disorders, and sleep apnea—and are associated with fatigue, mood disorders, and impaired cognitive function. However, the literature on sleep dysfunction in SAH remains sparse, heterogeneous, and largely descriptive. Almost all studies relied on self-reported questionnaires rather than quantitative sleep studies. In contrast, the literature on moderate to severe TBI is more robust, featuring standardized tools, longitudinal designs, and interventional studies. This discrepancy underscores a critical knowledge gap in SAH research and highlights the need for standardized, multimodal sleep assessments in this population. Given the bidirectional relationship between sleep and neuroplasticity, addressing sleep dysfunction may represent a modifiable target to enhance cognitive recovery.

Cognitive impairments are among the most disabling sequelae of SAH, affecting up to 71% of survivors even one year after the event. Memory, executive function, and attention are the most affected domains, and these deficits often persist despite “favorable” outcomes on conventional scales. Importantly, cognitive dysfunction—rather than physical disability—is frequently the primary barrier to returning to work and fully reintegrating into daily life. Yet cognitive outcomes remain under-assessed in both clinical practice and research. The tools used to evaluate cognitive function in SAH survivors vary widely, ranging from brief clinical scales such as the MoCA and MMSE to more comprehensive neuropsychological batteries. To date, studies on cognitive dysfunction following SAH are largely descriptive, offering limited insight into potential therapeutic interventions. Given the bidirectional relationship between sleep and cognition, routine assessment of sleep quality and targeted interventions to improve sleep hygiene may offer a promising avenue for enhancing cognitive recovery. Emerging evidence suggests that disrupted sleep architecture may contribute to the high prevalence of cognitive deficits observed in SAH survivors.

Fatigue is a central and overlapping symptom associated with both sleep dysfunction and cognitive impairment following SAH. It affects more than half of survivors and is strongly linked to reduced quality of life, depression, and impaired executive functioning. Despite its prevalence and impact, fatigue is rarely captured by traditional outcome measures and remains poorly understood in the context of SAH.

Little is known about the underlying pathophysiology and disease mechanisms driving prolonged post-SAH cognitive dysfunction, sleep disturbances, and fatigue. These symptoms are currently poorly quantified, with unclear clinical phenotypes and a lack of studies on potential risk factors. It is plausible that multiple mechanisms—alongside pre-SAH comorbidities and social determinants of health—contribute to the clinical syndromes of post-SAH cognitive and sleep dysfunction. Emerging evidence suggests that neuroinflammation may serve as a mechanistic bridge between acute brain injury and long-term cognitive and sleep disturbances. Inflammatory cytokines can disrupt sleep architecture and impair neuroplasticity, potentially contributing to persistent cognitive deficits. While animal models have shown that targeting neuroinflammation can improve cognitive outcomes, clinical translation remains limited. Future research should explore whether early modulation of neuroinflammatory pathways can mitigate long-term sequelae in SAH survivors.

This review identifies several critical gaps in the current literature. There is currently no consensus on how to assess sleep or cognitive dysfunction in SAH survivors, limiting comparability across studies and hindering clinical translation. Most existing studies are cross-sectional and descriptive; longitudinal research is needed to track symptom evolution and recovery trajectories. Unlike in TBI, no studies have tested interventions for sleep or cognitive dysfunction in SAH survivors. Traditional outcome measures fail to capture the lived experiences of SAH survivors. Future research must expand clinical trial endpoints beyond global functional outcomes to include patient-centered measures of sleep and cognitive health and prioritize the identification of modifiable risk factors and treatment strategies.

This scoping review has several limitations. The heterogeneity of the studies did not allow for a meta-analysis or quantitative synthesis. Additionally, the literature on sleep, cognitive dysfunction, and SAH survivorship is sparse, with most studies having small sample sizes and the usage of non-standardized tools. There is also the risk of publication bias for patient-reported outcomes like sleep quality, selection bias in cognitive testing, as well as self-report overestimation of prevalence in subjective outcome measures. Although moderate to severe TBI was used as a comparison to SAH, the differences between these diseases may lead to variations in the trajectories of patient recovery. It is reasonable to assume that there is a selection bias when it comes to cognitive assessments. Some SAH survivors are left in a condition where they are unable to speak or meaningfully participate in a cognitive assessment. This would lead to a selection bias against the most severe patients since they may be unable to meaningfully participate in the assessments. Additionally, the most severe patients may have even passed away while in the hospital and, therefore, not be captured in follow-ups. However, there are additional global assessments that can be performed, such as the mRS, that can include more severe patients.

## 5. Conclusions

Clinicians should be aware that favorable physical recovery does not equate to full recovery. Routine screening for sleep and cognitive dysfunction—using both qualitative and quantitative tools—should be integrated into post-SAH care. Early identification and targeted rehabilitation may improve long-term outcomes and quality of life. Moreover, clinical trials in SAH should expand their endpoints beyond global functional scales to include cognitive, emotional, and sleep-related outcomes.

## Figures and Tables

**Table 1:** Sleep dysfunction in the acute, subacute, and chronic phases of SAH recovery.

Author/Year	Sample Size, Key Population Characteristics & Study Design	Main Outcomes/Instruments Used
Acute Phase (0–3 Months)		
Byun et al. 2020 [53]	30, aSAH & naSAH, Hunt & Hess (H&H) 2–5, Cross-sectional	73% reported poor quality of sleep PSQI 27% reported daytime sleepiness [ESS] 41% experienced short (<7 h) or long (>9 h) sleep duration [Actigraphy] Self-efficacy correlates with nighttime sleep quality but not daytime sleepiness
Byun et al. 2022 [44]	30, aSAH & naSAH, H&H 2–5, Cross-sectional	33% reported difficulties falling asleep, waking after sleep onset, too much/too little sleep [Structured Interviews] 8% reported insufficient, poor quality of sleep 27% reported restlessness, daytime sleepiness during the first 3 months 15% reported that disturbances were worse at discharge but improved over time
Subacute Phase (3–12 Months)		
Brand et al. 2015 [46]	21 aSAH + 21 controls 5–9 months post-surgery, H&H 2–5, Cross-sectional	Subjective sleep (higher insomnia) was reported to be worse in aSAH patients than healthy controls ISI Objective sleep parameters were similar to controls [Overnight EGG] Psychological and cognitive deficits persist months after SAH
Ecker et al. 2024 [45]	70 at 3 months and 39 at 12 months post aSAH/naSAH and ICH, NIHSS 0–7, Retrospective cohort	26% had sleep disturbances (falling asleep, waking up, daytime sleepiness, pain) at 3 months, and 28% had sleep disturbances at 12 months [Neuro-QoL Short Form Sleep Disturbance] Sleep disturbances at 12 months were related to a premorbid mRS score No sustained relationship between preclinical/clinical factors and sleep disturbances post-stroke
Chronic Phase (>12 Months)		
Schuiling et al. 2005 [28]	83 aSAH & naSAH, 0–5 Modified Rankin Scale (mRS), Prospective, Assessment: 1–3.4 years after SAH	34% experienced severe sleep dysfunction (25% initiating, 31% maintaining, 28% returning, 31% tiredness, 6% excessive sleepiness during the day) SDL Sleep dysfunction is associated with reduced quality of life per the ESS Sleep monitoring showed severe sleep fragmentation, apnea, restless leg syndrome in 19 of 20 patients per PSG
Kreitschmann-Andermahr et al. 2007 [49]	40 aSAH & naSAH, Glasgow Outcome Scale (GOS) III-V, Observational cohort Assessment: 1 + year after SAH	Persistent impairments in QoL are correlated to depression/sleep disturbances (high Nottingham Health Profile Sleep Subscale score) [SF-36 and NHP Sleep Subscale] 27.5% reported mild to moderate (Beck Depression Index (BDI) 11–17) depressive symptoms Clinically relevant (BDI > 17) depression diagnosed in another 10% ACTH levels may predict NHP Sleep Subscale scores
Hütter et al. 1995 [51]	58 aSAH & naSAH, GOS I-II, Retrospective Assessment: 1–5 years after SAH	In “favorable outcome” SAH patients, quality of life reduced by sleep in 47% through the presence of tiredness and fatigue [Neuropsychological Examination]
Vetkas et al. 2013 [54]	114 aSAH, mRS 0–4, Retrospective Assessment: 1–10 years after SAH	26% reported sleep disturbances per the SF-36 46% of patients with sleep disturbances reported insomnia 47% of patients with sleep disturbances reported fatigue per the EST-Q Fatigue was independently related to depression and mental health components of the SF-36 scale
Sonesson et al. 2018 [48]	113 (93 aSAH and 20 SAH of unknown cause), GOS V, Retrospective cohort Assessment: 20–28 years after SAH	26% reported sleep disturbances per the RNL Supplement] 18% reported sleep initiating problems and 18% reported problems maintaining sleep per the RNL Supplement Sleep disturbances are correlated with lower psychological well-being (higher rates of depression, anxiety, etc.) Reintegration difficulties persist 20+ years after
Katzan et al. 2020 [52]	2213 (1412 ischemic stroke, 384 transient ischemic attack, 212 intracerebral hemorrhage, 205 aSAH/naSAH), mRS 0–2, Retrospective cohort Assessment: 3–5 years after event	31.2% of SAH participants had positive PROMIS sleep disturbance score 25% of SAH patients scored positive for insomnia. Rates of insomnia are higher in SAH than in ischemic stroke, intracerebral hemorrhage, transient ischemic attack patients per the ISI 45.1% SAH patients screened positive for sleep apnea (SAPS score), while the other three populations reported rates of 52.8–63.8% per the Sleep Apnea Probability Scale (SAPS) 29.7% of SAH patients reported <6 h in sleep duration 42.6% of SAH patients screened positive for fatigue [PROMISFatigue]
Ecker et al. 2022 [50]	73 (47 intracerebral hemorrhage, 26 aSAH/naSAH), mRS 1–5, Prospective Assessment: 1–7 years after SAH	Of 41 patients discharged to a destination other than home, 37% of participants reported sleep disturbances (falling asleep, waking up, daytime sleepiness, pain) and 49% reported fatigue per the Neuro-QoL Short Form Sleep Disturbance Of 44 patients with a 3-month mRS score of 3–5, 41% reported sleep disturbances and 55% reported fatigue
Yang et al. 2024 [47]	86 aSAH, mRS 0–2, Retrospective Assessment: 10–21 years after SAH	51.2% favorable outcome SAH survivors reported sleep problems per the PSQI Sleep dysfunction is associated with lower quality of life measured by EQ-5D

**Table 2:** Cognitive Dysfunction in the Acute, Subacute, and Chronic Phase of SAH Recovery.

Author/Year	Sample Size, Key Population Characteristics & Study Design	Main Outcomes
Acute Phase (0–3 Months Post-SAH)		
Mayer et al. 2002 [68]	113, aSAH, H&H 1–5, Prospective Cognitive Assessment at 3 months.	36% had global mental impairment per the TICS. The most common impairments were in verbal (42%) Motor Functioning (39%), and Reaction time (28%).
Wang et al. 2024 [69]	177, aSAH, H&H 1–5, Prospective Case-Controlled Cognitive Assessment at 2 months	78.43% of aSAH patients developed cognitive impairment compared to 16% in the control group.
Geraghty et al. 2020 [70]	105, aSAH, H&H1–5, Retrospective Cognitive Assessment prior to discharge	56.2% had a MoCA < 22(Mild impairment). 48.7% of patients with a good functional outcome mRS (0–2) still had cognitive impairment.
Rowland et al. 2021 [71]	27, aSAH H&H1–3, Prospective Cognitive Assessment at greater than 2 years	Almost 50%were cognitively impaired at 3 months, the most impacted domains were executive function processing speed, and non-verbal skills.
Kreiter et al. 2002 [72]	113, aSAH, H&H 1–5, Prospective Cognitive assessment at 3 months	>40% had verbal and motor functioning impairments. 25% had visual-spatial functioning impairment.
Mahajan et al. 2014 [73]	66, aSAH, H&H 1–2, Clinical Trial Cognitive Assessment at 1–13 days	Cognitive dysfunction (HMSE ≤ 23) was observed in 24 h post-op: 8 control patients, 12 in the interventional Group- using propofol as a neuroprotective agent. At discharge: there was no difference in cognitive dysfunction prevalence (5 in each group). Both groups showed a decline in cognition at 24 h followed by improvement at discharge. Propofol neuroprotection did not significantly improve cognitive outcomes.
Hütter et al. 1998 [74]	51, aSAH & naSAH, Fisher 1–3 Cross-Sectional Cognitive Assessments at 72 h, at discharge (14–28 days), 3 months	Severity of SAH (Fisher score) is the most important factor related to cognitive dysfunction. 73% of aSAH had significant deficits in figural short-term memory per the Benton Test as compared to 50% in the SAHs of unknown origin. 55% of aSAH had impairments in verbal long-term memory (per the IST Memory Scale) as compared to 33% in the SAHs of unknown origin. Frontal hematoma, intraventricular hemorrhage, and acute hydrocephalus were also associated with cognitive deficits.
Taufique et al. 2016 [8]	724, aSAH & naSAH, H&H 1–5, Prospective Cognitive Assessments pre-op, 24 h post-op, and at discharge	35% reported poor quality of life poor QOL was associated with difficulty concentrating (54%), cognitive dysfunction (53%), depression (68%), and inability it return to work at previous level (91%).
Rautalin et al. 2020 [75]	85, aSAH, mRS 0–2 Prospective Tested at Discharge and 3 months	95% of aSAH patients self-reported cognitive or emotional complaints that caused trouble with ADLs.
Haug Nordenmark et al. 2019 [76]	51, aSAH, WFNS 1–5 Prospective Assessed During Hospitalization	Acute cognitive deficits were frequent, especially in attention and memory. Poor WFNS grade and hydrocephalus predicted worse acute cognitive performance. Early dysfunction suggested risk for long-term impairment.
Wong et al. 2016 [77]	74, aSAH, WFNS 1–5 Prospective Assessed During Hospitalization	Early deficits in attention, memory, and executive function were common. Cognitive impairment correlated with poorer functional status (mRS, IADL). Suggests early testing can identify patients at risk for long-term dysfunction.
Wong et al. 2012 [78]	90, aSAH, WFNS 1–5 Prospective Assessed at 3 months	Cognitive impairment prevalence 73% at 3 months. Risk factors included age, WFNS grade, and delayed ischemia. MoCA scores correlated with functional outcomes.
Mahajan et al. 2012 [79]	100, aSAH, WFNS 1–5 Prospective Randomized Trial Assessed within 3 months	No major difference in global cognitive outcomes between anesthetic groups. Subtle domain differences: desflurane associated with slightly better attention scores. Cognitive dysfunction remained common postoperatively regardless of anesthetic choice.
Esmael et al. 2020 [80]	40, aSAH, WFNS 1–5 Prospective Assessed within Hospitalization-3 months	Elevated mean flow velocities predicted later cognitive impairment. Early TCD is useful for identifying patients at risk.
Lara-Angulo et al. 2019 [81]	84, aSAH, WFNS 1–5 Prospective Assessed within Hospitalization	The Modified Fisher Scale could be a predictor of severe cognitive impairment in aSAH survivors. Patients with good functional outcomes per the mRS still experience significant cognitive impairment.
Acute & Subacute Phases (0–12 Months (Post-SAH)		
Kälin et al. 2025 [82]	128, SAH, WFNS 1–5, Prospective Cognitive Assessment at 11–35 days, 6 months	The hydrocephalus group had higher odds (OR = 2.76) of experiencing cognitive decline during hospitalization. Hydrocephalus was not predictive of cognitive decline.
Bründl et al. 2018 [83]	21, aSAH & naSAH, H&H 1–3, Prospective Cognitive Assessment at 3, 6, 12 months	In the acute phase, CSF Neuropeptide Y (NPY) significantly correlated with worse scores in depression, anxiety and other psychological domains (ISR score) and in SF-36.
Khosdelazad et al. 2024 [84]	58, aSAH & naSAH, WFNS 1–5, Prospective Cognitive Assessment at 5 months	aSAH patients showed significant impairments in: Psychomotor speed, executive control, social cognition, memory per the VRT battery. anSAH patients showed significant impairments in psychomotor speed and executive control per the TMT-A&B.
Dronkers et al. 2025 [85]	325, naSAH, WFNS 1–5, Prospective Clinical Assessments at 6 months	89% of patients were mRS 0–2 Independent of bleed location ability to completely return to work was below 50%. 84% of participants with non-perimesencephalic naSAH reported residual symptoms: 23% had anxiety. 21% had depression. 68% had fatigue. 54% had concentration difficulties. 50% had forgetfulness.
Hedlund et al. 2011 [86]	83, aSAH, WFNS 1–5, Prospective Interviewed 12 mo. pre-bleed & 7 months post	41% of patients exhibited symptoms of depression and/or PTSD 7 months after SAH. Patients with a lifetime history of psychiatric disorders were significantly more likely to develop post-SAH depression or PTSD; associated with lower return-to-work rates.
Haug et al. 2007 [87]	32, aSAH, H&H 1–5, Prospective Multiple Cognitive assessments up to 1 year	Cognitive recovery is domain-specific and time-dependent: Motor and psychomotor functions improved within the first 6 months per the FAB. Verbal memory showed significant improvement only between 6 and 12 months, per the ROCF and VLMT. Attention remained impaired throughout the year per the DSF, WMS-R, VLMT.
Galea et al. 2017 [88]	146, aSAH, WFNS 1–5, Clinical Trial Cognitive Assessment at 6 months	41% had a good recovery 6 months per GOS-E.
Jorna et al. 2024 [89]	38, aSAH & naSAH, WFNS 1–5, Prospective Cognitive Assessment at 5 months	Significant Cognitive impairments in psychomotor speed (TMT-A/B, VTS RT), working memory (DSF/DSB), memory (15WT), executive control (TMT-B, VTS RT), social cognition (FEEST).
Powell et al. 2002 [90]	52, aSAH, WFNS 1–2, Prospective Cognitive Assessment at 3 & 9 months	Cognitive Dysfunction At 3 months: Immediate prose recall: 36.4% below 10th percentile. Delayed prose recall: 29.5% below 10th percentile. Backward digit span: 23% below 10th percentile. At 9 months: Immediate prose recall: 34.1% below 10th percentile. Delayed prose recall: 18.2% below 10th percentile. Backward digit span: 8.9% below 10th percentile.
Wong et al. 2014 [91]	108, aSAH, mRS 0–2 Prospective Assessed at discharge & 12 months	Early MoCA impairment predicted poor functional outcome at 1 year. Even patients with “good” mRS at discharge showed cognitive deficits. MoCA was more sensitive than mRS in detecting early dysfunction.
Dey et al. 2018 [92]	51, aSAH, WFNS I-II Prospective Assessed at 6 months	Despite good neurological recovery, ~40% had cognitive impairment at 6 months. Deficits mainly in attention and executive function. Functional outcome underestimated cognitive burden.
Shen et al. 2018 [93]	152, aSAH, All grades Prospective Assessed at 6 months	Mild cognitive impairment occurred in ~30% at 6 months. Risk factors included age, hypertension, and higher WFNS grade. Executive dysfunction was the most frequent deficit.
Wong et al. 2009 [94]	40, aSAH & naSAH, All grades Prospective Assessed between 6–12 months	Cognitive dysfunction correlated with reduced cholinergic activity (measured via biomarkers). Memory and attention deficits were most pronounced. Suggests cholinergic dysfunction may underlie persistent cognitive impairment.
Wong et al. 2013 [95]	80, aSAH, mRS 0–2 Prospective Assessed between 3–6 months	MoCA detected significantly more cognitive deficits than MMSE. Executive and attention impairments were often missed by MMSE. MoCA is recommended as a superior screening tool in aSAH.
Saciri et al. 2002 [96]	59, aSAH, WFNS 1–5 Prospective Assessed within 6 months	Early rehabilitation improved functional recovery, but cognitive deficits persisted. Memory and executive dysfunction remained common at 6 months. Rehabilitation enhanced independence but did not fully restore cognition.
Ma et al. 2021 [97]	126, aSAH, WFNS 1–2 Multicenter Retrospective Assessed between 6–12 months	Cognitive impairment occurred in ~30% despite low clinical severity. Risk factors included age, hypertension, and surgical approach. Deficits mainly in memory and executive domains.
Barrozo et al. 2019 [98]	aSAH, WFNS 1–5 Prospective Assessed within 6 months	High prevalence of depression, anxiety, and cognitive impairment. Neuropsychiatric dysfunction correlated with poorer functional recovery. Highlights need for integrated cognitive and psychiatric screening.
Hasan et al. 2020 [99]	30, aSAH, WFNS 1–5 Prospective Assessed at Discharge, 3 & 12 months	Cognitive deficits persisted across time points, especially in executive and memory domains. Functional recovery (mRS) underestimated cognitive dysfunction. Longitudinal testing highlighted evolving deficits not captured by single assessments.
Chronic Phase (>12 Months After SAH)		
Rass et al. 2024 [65]	177, aSAH & naSAH, H&H grade 1–3, Prospective Cognitive Assessment at 1 year	71% of patients had cognitive deficits in at least one domain: Memory: 51%, Executive function: 36%, Attention: 21% 60% of participants with mRS = 0 had cognitive deficits. 34% of working-age individuals were able to return to their previous job.
Tölli et al. 2018 [100]	35, aSAH, Fisher 2–4, Prospective Cognitive Assessments at 12 months	76% experienced cognitive impairment at 3 months, 70% at 6 months and 59% at 12 months. SAH recovery was slower than TBI but continued after 6 months.
Rass et al. 2020 [101]	43, aSAH, H&H 1–5, Prospective Cognitive Assessments at 12 months	22% were cognitively impaired per MMSE. 26% had executive function impairment per the FEDA distractibility questionnaire. 30% reported fatigue per the FEDA fatigue questionnaire.
Säveland et al. 1986 [102]	31, aSAH, H&H 1–5, Retrospective Cognitive Assessment at 1 year.	41% of patients had a good physical recovery. 33% had a favorable outcome when cognitive and psychosocial impairments were considered. 19% of those with good recovery had severe cognitive and psychosocial incapacitation.
Ørbo et al. 2008 [103]	42, aSAH, H&H 1–3, Prospective Cognitive Assessment 1 year. post-surgery	93% of patients had at least one cognitive score below normal- most impairments in memory, Executive Function, and information processing. Fisher Grade was the only significant predictor of Cognitive Outcome.
Hårdemark et al. 1989 [104]	21, aSAH, H&H 1–5, Prospective Cognitive Assessment at 2 years.	100% of patients demonstrated some cognitive impairment on one or more tests performed. Elevated CSF S-100 protein levels during days 2–8 post-SAH were significantly associated with Poor functional outcomes (Glasgow Outcome Scale).
Nozaki et al. 2002 [105]	17, aSAH, WFNS 1–4, Observational Cognitive Assessments at 4+ years.	47% were cognitively impaired per MMSE. Cognitively impaired individuals had a significantly higher relativization ratio. AH severity (WFNS grade) also correlated with both MMSE scores and the dilation ratio, suggesting that more severe hemorrhages were linked to worse cognitive and cholinergic outcomes.
Kreitschmann-Andermahr et al. 2004 [106]	40, aSAH, H&H 1–4, Observational Cognitive Assessment at 1–6 years. post bleed	55% of patients had some form of hypopituitarism. −32.5% had isolated corticotroph deficiency −12.5% had isolated severe growth hormone deficiency (GHD) −7.5% had combined corticotroph and GHD. Concluded that neuroendocrine dysfunction may contribute to long-term cognitive deficits after SAH.
Storey et al. 2025 [107]	12, aSAH & naSAH, Retrospective Qualitative Service Analysis Interviewed at 7–30 months Post bleed	Cognitive impairments (e.g., memory, attention, concentration) were barriers to engaging with support. Participants described difficulties retaining information, following group conversations, and managing fatigue.
Zabyhian et al. 2018 [108]	53, aSAH, mRS 0–2, Prospective Cognitive Assessment at 9–48 months	56.6% of patients exhibited cognitive impairment, with 22.6% being severe.
Sonesson et al. 1987 [55]	93, all aneurysmal, H&H 1–3, Retrospective Cognitive Assessment at 12–103 months	Cognitive impairment: Moderate dysfunction: 32% Late Surgery (LS) vs. 35% Early Surgery (ES) (impaired on 2–3 tests) Marked dysfunction: 34% (LS) vs. 29% (ES) (impaired on 4–5 tests) Severe dysfunction: 18% in both groups (impaired on 5+ tests). Memory impairment was most common with 89% in the LS group per the CMI/CMII, BVR, and MFD.
Ljunggren et al. 1985 [109]	40, all aneurysmal, H&H 1–3, Cross-Sectional Cognitive Assessment at 14–84 months	30% with moderate impairment (impaired on 2–3 tests). 53% were found to have marked impairment (impaired on 4–5 tests). 83% of patients had memory impairments per the CMI/CMII, BVR, and MFD.
Plata-Bello et al. 2017 [110]	12, aSAH & naSAH, Fisher score 1–3; no vasospasm, Cross-Sectional Cognitive Assessment sat 1 year	25% of SAH survivors showed visuospatial/visuospatial impairment.
Persson et al. 2019 [111]	18, non-traumatic SAH, H&H 1–5, Retrospective Cognitive Assessments at 7 years	61% of participants had Cognitive impairment (MoCA < 26). 11% had moderate impairment (MoCA < 17). 89% had delayed recall impairment. 56% had abstraction impairment. 39% had impairment in visuospatial tasks. 32% had attention impairments.
Springer et al. 2009 [112]	232, all SAH, H&H1–5, Prospective Assessed at 12 and 3 months	~37% had global cognitive impairment at 1 year. Predictors included older age, poor WFNS grade, and delayed cerebral ischemia. Cognitive impairment was strongly associated with reduced QOL and functional independence.
Wong et al. 2014 [113]	194, aSAH, mRS 0 Prospective Assessed 12+ months	Despite excellent functional recovery, ~40% had MoCA-detected cognitive impairment. Deficits were most common in executive function and memory domains. Highlights that “excellent” mRS outcomes can mask persistent cognitive dysfunction.
Stienen et al. 2014 [114]	92, aSAH, WFNS I-Vm Prospective Assessed after 12 months	Delayed cerebral ischemia (DCI) was a strong predictor of long-term cognitive impairment. Memory and executive dysfunction persisted despite good mRS outcomes. DCI explained variance in neuropsychological deficits beyond initial clinical grade.
Latimer et al. 2013 [115]	23, aSAH, “Stable outcome” Retrospective Assessed after 12 months	Both clipping and coiling groups showed persistent cognitive deficits at ≥1 year. No significant difference between treatment modalities. Memory and executive dysfunction are common, impacting daily functioning.
Beeckmans et al. 2020 [116]	35, aSAH, All grades Prospective Assessed after 12 months	Both clipping and coiling groups had persistent cognitive deficits at 1 year. No significant difference between treatment modalities. Memory and executive dysfunction were most common.
Schweizer et al. 2012 [117]	32, aSAH, mRS 0, Prospective Assessed after 12 months	MoCA identified cognitive impairment in ~40% despite excellent mRS. MMSE failed to detect most executive/memory deficits. Functional scales underestimated cognitive burden.
Walter et al. 2020 [118]	104, aSAH, WFNS 1–5 Prospective Assessed after 12 months	NAB detected subtle deficits in executive and memory domains. Strong correlation between NAB scores and quality of life. NAB is considered more comprehensive than rapid screening tools.
Han et al. 2022 [119]	336, aSAH & naSAH, WFNS 1–5 Prospective Assessed after 12 months	Hp2–2 phenotype associated with worse cognitive outcomes. Memory and executive dysfunction more common in Hp2–2 carriers. Genetic variation influenced long-term neurological recovery.
Eagles et al. 2019 [120]	337, aSAH, WFNS 1–5 Prospective Assessed after 12 months	DCI strongly predicted long-term cognitive impairment. Memory and executive dysfunction persisted despite good mRS outcomes. Functional scales underestimated cognitive burden.
Hütter et al. 1995 [51]	58, aSAH & naSAH, WFNS 1–5 Prospective Assessed at ≥12 months	Cognitive deficits persisted in ~40% at 1 year. Strong correlation between cognitive impairment and reduced quality of life. Functional recovery did not guarantee cognitive recovery.
Ogden et al. 1997 [121]	123 aSAH & SAH, WFNS 1–5 Prospective Assessed between 4–7 years	Persistent cognitive deficits in memory and executive domains at 4–7 years. Psychosocial dysfunction (employment, social reintegration) common despite neurological recovery. Long-term quality of life significantly reduced.
Ross et al. 2013 [122]	214, aSAH, WFNS 1–5 Prospective Asssessed at ≥12 months	Patient-reported cognitive complaints persisted despite good mRS outcomes. Fatigue, memory problems, and executive dysfunction were most frequently reported. Highlights importance of including patient-reported outcomes in long-term follow-up.
Bernardes Camilo Castilho de Avellar et al. 2016 [123]	44, aSAH & naSAH, mRS 0–2 Prospective Assessed at ≥12 months	~40% had cognitive impairment despite good functional recovery. Deficits most common in executive function and memory. Functional scales underestimated cognitive burden.
Wenneberg et al. 2022 [9]	62 at 1 year, 54 at 3 years, aSAH, WFNS 1–5 Prospective	Cognitive impairment persisted in ~30% at 5 years. Quality of life and psychosocial functioning reduced long-term. Predictors included age, WFNS grade, and delayed ischemia.
Gaastra et al. 2022 [124]	884, aSAH, WFNS 1–5 Retrospective Mean Assessment at 7 years.	Cognitive impairment persisted in ~40% at 7 years. Executive and memory deficits most common. Long-term dysfunction correlated with initial WFNS grade and complications.
Krajewski et al. 2014 [125]	13 aSAH, 14 naSAH, WFNS 1–5 Prospective Assessed at ≥12 months	aSAH patients had the most severe long-term cognitive deficits. Perimesencephalic SAH showed milder impairment. Incidental aneurysm patients had near-normal cognition.
Hütter et al. 2017 [126]	4, aSAH, WFNS 1–5 weeks. Hydrocephalus Case Studies Assessed post shunting and 2–5 years.	Shunting improved short-term neurobehavioral function. Long-term follow-up showed persistent cognitive deficits despite hydrocephalus treatment. Memory and executive dysfunction remained common.
Wong et al. 2013 [127]	168, aSAH, WFNS 1–5 Prospective Assessed at ≥12 months	~40% had domain-specific deficits, such as executive dysfunction and attention at 1 year. Memory and executive dysfunction most frequent. Risk factors included age and delayed cerebral ischemia.
Sheldon et al. 2012 [128]	24, aSAH, WFNS 1–5 Prospective Assessed at ≥12 months	Free recall memory significantly impaired compared to controls. Recognition memory relatively preserved. Suggests specific hippocampal/temporal dysfunction after aSAH.
Santos Cechi et al. 2025 [129]	31, aSAH, WFNS 1–5 Prospective Assessed at ≥12 months	Visuospatial deficits common at 1 year post-clipping. Deficits correlated with surgical approach and aneurysm location. Functional recovery underestimated visuospatial dysfunction.
Sanchez et al. 2021 [130]	10, aSAH, WFNS 1–5 Pilot Study Assessed at mean of 3–5 years.	Long-term survivors showed persistent spatial reference memory deficits. Deficits correlated with quality of life measures. Highlights utility of novel paradigms for detecting subtle dysfunction.

## Data Availability

The original contributions presented in this study are included in the article/Supplementary Material. Further inquiries can be directed to the corresponding author(s).

## Supplementary Materials

The following supporting information can be downloaded at https://www.mdpi.com/article/10.3390/jcm15031002/s1. Table S1: Literature Search Strategy, Table S2: SAH Phase x Type of Sleep Disturbance Schematic Matrix, Table S3: Cognitive Tool x Phase Schematic Matrices, Table S4: Summary of sleep tools used in SAH and TBI studies, Table S5: Cognitive Assessment Tools Used in SAH Studies, Text: Discussion on MoCA Variants, Table S6: MoCA Variants, Table S7: Non-Standard Acronyms and Abbreviations. References [157,158,159,160,161,162,163,164] are cited in the supplementary materials Table S6.
