# Supplementary material for: Sleep and Cognitive Dysfunction in Subarachnoid Hemorrhage: A Scoping Review
Source: J Clin Med. 2026 Jan 26;15(3):1002. doi: 10.3390/jcm15031002 (PMC12898122; doi:10.3390/jcm15031002)
Supplement: Supplementary file 1 [file jcm-15-01002-s001.zip › jcm-3883751-supplementary.pdf]

[Supplemental Table S1: Literature Search Strategy

Database	Search Topic	Search String (Simplified)	Filters Applied	Results
**PubMed**	SAH + Sleep	("Subarachnoid Hemorrhage"[MeSH Terms] OR "Subarachnoid Hemorrhage"[Title/Abstract] OR "subarachnoid haemorrhage"[Title/Abstract] OR "SAH"[Title/Abstract]) AND ("Sleep"[MeSH Terms] OR "Sleep"[Title/Abstract])	English, Humans, Adults, 1980–2025	68
**PubMed**	SAH + Cognitive Dysfunction	("Subarachnoid Hemorrhage"[MeSH Terms] OR "Subarachnoid Hemorrhage"[Title/Abstract] OR "subarachnoid haemorrhage"[Title/Abstract] OR "SAH"[Title/Abstract]) AND ("Cognitive Dysfunction"[MeSH Terms] OR "Cognitive Dysfunction"[Title/Abstract] OR "cognitive impairment"[Title/Abstract])	English, Humans, Adults, 1980–2025	155
**PubMed**	TBI + Sleep	("Traumatic Brain Injury"[MeSH Terms] OR "TBI"[Title/Abstract] OR "brain injuries, traumatic"[Title/Abstract] OR "moderate TBI"[Title/Abstract] OR "severe TBI"[Title/Abstract] OR "moderate-to-severe TBI"[Title/Abstract] OR "moderate to severe TBI"[Title/Abstract]) AND ("Sleep"[MeSH Terms] OR sleep[Title/Abstract] OR "Sleep Disorders"[MeSH Terms])	English, Humans, Adults, 1980–2025	122
**Embase** (Ovid)	SAH + Sleep	('subarachnoid hemorrhage'/exp OR 'subarachnoid hemorrhage':ti,ab OR	English, Humans, Adults, 1980–2025	475

		'subarachnoid haemorrhage':ti,ab OR sah:ti,ab) AND ('sleep'/exp OR sleep:ti,ab)		
**Embase** (Ovid)	SAH + Cognitive Dysfunction	('subarachnoid hemorrhage'/exp OR 'subarachnoid hemorrhage':ti,ab OR 'subarachnoid haemorrhage':ti,ab OR sah:ti,ab) AND ('cognitive dysfunction':ti,ab OR 'cognitive impairment':ti,ab)	English, Humans, Adults, 1980–2025	298
**Embase** (Ovid)	TBI + Sleep	('traumatic brain injury'/exp OR 'traumatic brain injury':ti,ab OR 'moderate tbi':ti,ab OR 'severe tbi':ti,ab OR 'moderate-to-severe tbi':ti,ab) AND ('sleep'/exp OR sleep:ti,ab OR 'sleep disorder*':ti,ab)	English, Humans, Adults, 1980–2025	205
**Web of Science**	SAH + Sleep	TS=("subarachnoid hemorrhage" AND sleep)	English, Article Type, 1980–2025	157
**Web of Science**	SAH + Cognitive Dysfunction	TS=("subarachnoid hemorrhage" AND "cognitive dysfunction")	English, Article Type, 1980–2025	338
**Web of Science**	TBI + Sleep	TS=("traumatic brain injury" AND sleep AND ("moderate" OR "severe"))	English, Article Type, 1980–2025	525

Supplemental Table S2: SAH Phase x Type of Sleep Disturbance Schematic Matrix

	Type of Sleep Disturbance								
SAH Phase	Insomnia	Hypersomnia	Difficulty initiating/maintaining /returning to sleep	Too little or too much sleep	Sleep fragmentation	Restless Leg Syndrome	Sleep Apnea	Fatigue	General Complaints
Acute (0-3 months)	73% [53]	27% [53]	33% [44]	8-41% [44,53]	No Data	No Data	No Data	No Data	No Data
Subacute (3-12 months)	No Data	26-28% [45,46]	26-28% [45,46]	26-28% [45,46]	No Data	No Data	No Data	No Data	No Data
Chronic (> 12 months)	25-46% [52,54]	6-47% [28,51]	18-37% [28,48,50]	29.70% [52]	95% [28]	95% [28]	45.1-95% [28,52]	42.6-55% [50,51,54]	26-51.2% [47,52,54]

Supplemental Table S3: Cognitive Tool x Phase Schematic Matrices

A.

Global Cognition	Tool Used (Proportion Below cut-off)									
SAH Phase	ACE-R	BIOCTO-39	BNIS	CDT	GPT	MMSE	MoCA	OMC/ OMCT	SBT	TICS
Acute (0-3 months)	No Data	No Data	No Data	No Data	44-48% [68]	53% cognitive dysfunction reported during hospitalization8	53.8-73% [70,75,78]	No Data	36% [68]	36% [68]
Subacute (3-12 months)	No Data	No Data	No Data	No Data	No Data	34–36% cognitively impaired on ≥1 domain at 3–9 months92	30-40% [92,93]	No Data	No Data	No Data
Chronic (> 12 months)	No Data	No Data	No Data	No Data	No Data	22% cognitively impaired [101]; 47% [105]; 37% at 12 months [14]; 56.6% with 22.6% severe [108]	40% cognitively impaired despite good functional outcome [113]; 40% cognitively impaired despite mRS = 0 [117]; 61% MoCA < 26 at long-term follow-up [111]	No Data	No Data	21% cognitively impaired at 12 months (TICS ≤30) [112]

B.

Executive Function, attention, and processing speed	Tool Used (Proportion Below cut-off)														
SAH Phase	CLOX	COWAT	DSB/F	FAB	FEDA	HOCOT	IST	Lawton IADL	RWT	RCFT/ ROCFT	SCWT	TMT-A	TMT-B	WCST/ MWCST	Zimmermann Battery (Task 1/2/3)
Acute (0-3 months)	No Data	No Data	"At three months [...] 23% [scored below the 10th percentile] on backwards digit span" [90]	No Data	No Data	No Data	aSAH 55% nSAH 33% [74]	Median 13 [8]	No Data	25-48% [68]	No Data	48-50% [68]	38-48% [68]	13-48% [68]	aSAH: 7-43% nSAH: 0-65% [74]
Subacute (3-12 months)	No Data	No Data	Reduced Digit Span Forward performance compared to healthy controls, while Digit Span Backward did not reach statistical significance [89]; 8.9% scored below the 10th percentile on Digit Span Backward at 9 months [90]	No Data	No Data	No Data	No Data	No Data	No Data	Visual memory was reduced at 3 months and showed a significant improvement from 3 to 6 months and from 6 to 12 months, with scores being at the expected normative mean 1 year after SAH [87]	No Data	“Cognitive impairments were found for both SAH groups relative to HC” [84], and “cognitive impairments were evident across all domains (psychomotor speed, memory, executive control, and social cognition)”	"Patients with aSAH performed significantly worse on psychomotor speed, executive control and social cognition when compared to HC" [84], and “cognitive impairments were evident across all domains (psychomotor speed, memory,	No Data	No Data

												[89]	executive control, and social cognition)” [89]		
Chronic (> 12 months)	No Data	No Data	No Data	No Data	26% executive impairment; 30% fatigue65	No Data	No Data	No Data	No Data	No Data	Executive inhibitory control impaired at 12 months, with 56.4% scoring below T40 and 48.7% below T30 on Stroop interference [103]	No Data	No Data	Executive dysfunction on set-shifting, with 37.1% impaired on WCST errors and 40.0% on perseverative responses (T < 40) [103]; executive function/psycho motor speed deficits present in 14% at 1 year [113]	No Data

C.

Memory, learning, processing	Tool Used (Proportion Below cut-off)														
SAH Phase	15WT	CAL CAP	CFQ	CMI/CMII	CVLT	RAVLT	RC/ RCF-R	SRB:1	SRT	SWR	VLMT	VMLT	VPA/ vPAL/ VAMPT	VRT	WMS-R
Acute (0-3 months)	No Data	24-48% [68]	No Data	No Data	48% [68]	No Data	31% [68]	No Data	No Data	No Data	No Data	No Data	No Data	No Data	22% [68,72]
Subacute (3-12 months)	"Cognitive impairments were evident across all domains (psychomotor speed, memory, executive control, and social cognition)" [89]; statistically significant reductions in verbal memory compared to healthy controls at 5 months post-ictus [84]	No Data	No Data	No Data	No Data	No Data	No Data	No Data	No Data	No Data	"The patients showed impairment in verbal memory after 3 months, with no significant change at 6 months after SAH. However, they significantly improved from 6 to 12 months." [87]	No Data	No Data	No Data	No Data
Chronic (> 12 months)	No Data	No Data	No Data	Memory impairment 89% [55]; 83% [109]	No Data	No Data	No Data	No Data	No Data	No Data	No Data	No Data	No Data	No Data	37% memory impairment [112]

D.

Visual and spatial processing and learning	Tool Used (Proportion Below cut-off)						
SAH Phase	BVD	BVR	BVRT	FRT	German Bourdon-Wiersma	JLOT	VSAT
Acute (0-3 months)	No Data	No Data	No Data	No Data	No Data	No Data	45-48% [68]
Subacute (3-12 months)	No Data	No Data	No Data	No Data	No Data	No Data	No Data
Chronic (> 12 months)	No Data	89% memory impairment [55]; 83% memory impairment [109]	No Data	No Data	No Data	No Data	No Data

E.

Emotion & Mood	Tool Used (Proportion Below cut-off)										
SAH Phase	BD/BDI	BI	BNT/ BGRT	BSI	CBT	CES-D	HADS	FEEST	MFD	RIES	RLCQ
Acute (0-3 months)	No Data	No Data	48-51% [68]	No Data	No Data	No Data	No Data	No Data	No Data	No Data	No Data
Subacute (3-12 months)	No Data	No Data	No Data	No Data	No Data	No Data	Depression and/or PTSD in 41% of patients; anxiety in 46% and depressive symptoms in 33% [86,98]	Reduced social cognition performance compared with healthy controls (SAH: 45.8 ± 5.6 vs 49.1 ± 4.9), statistically significant [89]	No Data	No Data	No Data
Chronic (> 12 months)	No Data	No Data	No Data	No Data	No Data	No Data	No Data	No Data	No Data	No Data	No Data

F.

Global measurements of outcome, health, quality of life	Tool Used (Proportion Below cut-off)								
SAH Phase	EQ-5D	GOS-E	GOS	LiSAT-11	MFI	MRS	SF-36	SIP	SWLS
Acute (0-3 months)	No Data	No Data	No Data	No Data	No Data	66.7% impaired despite mRS 0, 53.8% impaired despite mRS 0–2 [75]; 57% had severe cognitive impairment that was not reflected in the mRS in about half of the cases [76]	No Data	No Data	No Data
Subacute (3-12 months)	No Data	41% good recovery at 6 months [88]	No Data	No Data	No Data	40% cognitively impaired despite good functional outcome (mRS 0–2) [92]	Higher CSF neuropeptide Y levels correlated with poorer mental health–related quality of life on SF-36 [83]	No Data	No Data
Chronic (> 12 months)	No Data	No Data	41% good physical recovery; only 33% favorable when cognition included [102]	No Data	No Data	60% of patients with mRS = 0 still had cognitive deficits [101]; ~40% cognitively impaired despite excellent mRS [113,123]	Cognitive impairment at 12 months associated with worse SIP physical, psychosocial, and total scores [112]	No Data	No Data

G.

Other Multi-Domain	Tool Used (Proportion Below cut-off)						
SAH Phase	NART	RLAS-R/ LCS	RLS85	TT	VTS	WAB AQ & CQ	WASI-II
Acute (0-3 months)	No Data	No Data	No Data	15% [68]; aSAH 39% nSAH 28% [74]	No Data	No Data	No Data
Subacute (3-12 months)	No Data	No Data	No Data	No Data	Impaired psychomotor speed and executive control on reaction time measures (VTS) [89]	No Data	No Data
Chronic (> 12 months)	No Data	No Data	No Data	No Data	No Data	No Data	No Data

Supplemental Table S4. Summary of sleep tools used in SAH and TBI studies

Assessment Tool	Measurement	duration	Domain	Strengths	Limitations	Used In
**Sleep specific assessments**						
**Epworth Sleepiness Scale (ESS)** [44]	General daytime sleepiness	2-5 minutes	hypersomnia	● Quick to administer<br>● Widely validated for hypersomnia	● Limited scope	SAH/TBI
**Pittsburgh Sleep Quality Index (PSQI)** [28]	Sleep quality	5-10 minutes	sleep habits and experiences over a month	● Widely validated<br>● Comprehensive view of sleep habits	● May be subject to recall bias from subject	SAH/TBI
**Sleep Diagnosis Questionnaire (SDL)** [28]	Problems with sleep and wake	25-35 minutes	sleep disorders across 3 dimensions	● Multiple dimensions of sleep dysfunction covered	● Time-consuming	SAH
**PROMIS Sleep Disturbance** [52]	Sleep quality and depth	1-2 minutes	sleep quality and depth	● Quick to administer	● Lacks depth	SAH
**Insomnia Severity Index (ISI)** [52]	Severity of insomnia	3-5 minutes	severity and impact of insomnia	● Tailored to detailed facets of insomnia	● Limited to only insomnia	SAH/TBI
**Sleep Apnea Probability Scale (SAPS)** [52]	Likelihood of having obstructive sleep apnea	N/A	obstructive sleep apnea	● Screens for obstructive sleep apnea (OSA)	● Not a diagnostic tool	SAH
**NHP Sleep Subscale** [49]	Sleep quality and well-being	2-3 minutes	impact of sleep on overall well-being	● Links sleep to well-being<br>● Quick to administer	● Limited specificity	SAH
**Structured Interview** [53]	Subjective sleep quality and lived experience	N/A	individual's unique experience with sleep disturbances	● Captures subtle nuances of the lived experiences of patients	● Lacks standardization<br>● Time-consuming	SAH

**Polysomnography (PSG)** [28]	Physiological parameters	8-10 hours	Quantitative assessment of sleep disorders	● Gold standard for diagnosing sleep disorders	● Expensive<br>● Time-consuming	SAH/TBI
**Actigraphy [53]**	Body movements	3 days to 2 weeks	sleep and wakefulness	● Long-term monitoring of physiological aspects of sleep	● Time-consuming	SAH/TBI
**Quality of Life Questionnaires containing questions about sleep**						
**SF-36 [28]**	"Did you feel tired?"	< 1 minute	Presence of tiredness in daily life	● Quick to administer	● Non-specific and lacks detailed insight into sleep dysfunction	SAH/TBI
**Quality of Life in Neurological Disorders (Neuro-QoL)** [50]	Presence of sleep disturbances and fatigue	3-5 minutes	Disturbances to sleep cycle and fatigue during the day	● Assesses sleep in context of neurological health	● Non-specific and doesn't capture details of sleep quality	SAH
**EST-Q [54]**	Fatigue and insomnia	1-2 minutes	How easily one is fatigued, difficulty falling asleep, restless/disturbed sleep, waking up too early	● Quick to administer	● Limited scope	SAH
**RNL Supplement** [48]	Fatigue and tiredness	1-2 minutes	Need for daytime rest, fatiguability	● Quick to administer	● Not specific to sleep<br>● Limited detail	SAH

Supplemental Table S5: Cognitive Assessment tools used in SAH Studies

Instrument	Measures	Duration	Specificity
**Global Cognition**			
ACE-R	Attention, memory, verbal fluency, language, and visuospatial abilities	15-20 min	Global cognition
BIOCTO-39	Multi-domain cognitive functioning, including memory, attention, executive function, language, abstraction, and visuospatial processing.	15-20 min	Global cognition with emphasis on contextual memory and executive control.
BNIS	Higher cerebral functions including language, orientation, concentration, visuospatial skills, memory, affect, and self-awareness.	10-15 min	Global cognition with emphasis on executive and affective functioning
CDT	Visual-spatial abilities, executive function, and attention; useful for screening cognitive impairment	3-5 min	Global cognition with emphasis on executive and visuospatial functioning
GPT	Memory, orientation, and executive function through patient and informant input	5 min	Global Cognition
MMSE	Orientation, memory, attention, language, and visuospatial skills to screen for cognitive impairment	10-15 min	Global Cognition
MoCA	Multiple domains, including memory, attention, executive function, language, visuospatial skills, and orientation	10 min	Global Cognition: emphasis on executive function and attention

OMC/ OMCT	Orientation, memory, and concentration to screen for cognitive impairment	5-10 min	Global cognition with emphasis on verbal memory and attention
SBT	Orientation, memory, and concentration to screen for cognitive impairment	5-10 min	Global cognition with emphasis on attention and memory
TICS[90]	Global cognitive functioning including memory, attention, and language	10 min	Global cognition
**Executive Function, attention and processing speed**			
CLOX	Executive function (CLOX1) and constructional ability (CLOX2) via clock drawing	5-10 min	Executive functioning and visuospatial skills
COWAT	Verbal fluency, executive function, and cognitive flexibility	5 min	Language and executive functioning
DSB/F	Attention, short-term memory, and working memory	5-10 min	Working Memory and Executive Function
FAB	Executive functions including conceptualization, mental flexibility, motor programming, inhibitory control, and environmental autonomy	10 min	Executive Functioning
FEDA	Executive dysfunction via verbal fluency, inhibition, and working memory	5-10 min	Executive function and differential dementia diagnosis.
HOCOT	Complex reasoning, problem-solving, and metacognitive skills across disciplines	20-30 min	Executive function, abstract reasoning, and cognitive flexibility
IST	Decision-making, impulsivity, and executive function by assessing how individuals gather and use information before making choices	10-15 min	Executive Functioning and Decision-making
Lawton IADL	Ability to perform complex daily tasks such as using the phone, shopping, food preparation, housekeeping, and managing finances	10-15 min	Functional independence with emphasis on cognitive and executive functioning
RWT	Verbal fluency, executive function, and lexical retrieval through phonemic and semantic word generation tasks	5-10 min	Language and Executive Functioning
RCFT/ ROCFT	Visual memory, visuospatial skills, attention, planning, and executive function	45 min w. Delayed recall	Visuospatial memory and executive functioning
SCWT	Cognitive interference, attention, processing speed, and executive control	5-10 min	Executive function and cognitive flexibility
TMT-A	Visual attention, processing speed, and sequencing.	2-5 min	Attention and Processing Speed

TMT-B	Executive function, mental flexibility, and task switching	3-5 min	Executive Functioning
WCST/<br>MWCST	Executive function, cognitive flexibility, and problem-solving through rule-shifting tasks.<br>Modified: Executive function, particularly cognitive flexibility, problem-solving, and set-shifting	20-30 min<br>Modified: 15-20 min	Executive Functioning
Zimmermann Battery (Task 1/2/3)	Attention, processing speed, and executive function through computerized reaction time and decision-making tasks	15–20 minutes	Attention and Executive Functioning
**Memory, learning, processing**			
15WT	Episodic memory via recall of 15 words over multiple trials and delayed recall.	10-15 min	Verbal memory and retention
CALCAP	Reaction time, attention, working memory, and speeded information processing	Standard 20-25 min<br>Abbreviated 8-10 min	Processing speed, Attention, and working memory
CFQ	Self-reported frequency of everyday cognitive lapses (e.g., forgetfulness, distractibility)	10-15 min	Attention, memory, perception, and action slips
CMI/CMII	Experiential psychotherapy model for integrating unresolved emotional experiences	Varies	Memory reconsolidation, emotional processing, and trauma integration
CVLT	Verbal learning and memory including encoding, recall, and recognition	20-30 min	Verbal episodic memory
RAVLT	Verbal learning, immediate and delayed recall, recognition, and susceptibility to interference	20-30 min	Verbal Memory and Learning
RC/<br>RCF-R	Visuospatial construction, visual memory, attention, planning, and executive function	15-30 min	Non-verbal memory and visuoconstructional ability
SRB:1	Processing speed, attention, and visual scanning	5 min	Cognitive Processing efficiency and Attention
SRT	Implicit learning, motor skill acquisition, and procedural memory through reaction time to patterned stimuli	15-30 min	Implicit learning and motor sequencing
SWR	Semantic memory and lexical access through tasks that require retrieval of words based on meaning	5-10 min	Language and Semantic Processing

VLMT	Verbal learning and recognition memory using semantically unrelated word lists	5-10 min	Verbal Memory and Learning
VMLT	Verbal learning, recall, recognition, and memory strategies	20-30 min	Verbal memory and Executive Function
VPA/ vPAL/ VAMPT	Associative and episodic memory via recall of word pairs	5-10 min	Verbal Memory and Learning
VRT	Visual memory, spatial reasoning, and visual perception	15-20 min	Visual memory and cognitive processing
WMS-R	Verbal, visual, immediate, delayed, and working memory through multiple subtests	30-60 min	Memory (verbal, visual, working, immediate, delayed)
**Visual and spatial processing and learning**			
BVD	Visuospatial memory through recall and recognition of geometric designs	30-45 min	Visual and spatial processing
BVR	Visual memory, perception, and visuoconstructive skills.	10-15 min	Visual memory and spatial Processing
BVRT	Visual memory, visual perception, and visuoconstructive abilities through reproduction of geometric designs	15-30 min	Visual memory and visuospatial processing
FRT	Ability to recognize and match unfamiliar faces; sensitive to visuospatial and perceptual deficits	Short Form: 5–15 minutes (27 items) Long Form: 10–20 minutes (54 items)	Visual perception, face processing, and social cognition
German Bourdon-Wiersma	Visual perception, sustained attention, and vigilance through a dot cancellation task	10-15 min	Attention and Visual Scanning
JLOT	Visuospatial judgment and orientation perception	10-15 min	Spatial processing and parietal lobe function
VSAT	Visual scanning and sustained attention using visual cancellation tasks	6 min	Attention and Visuospatial processing
**Emotion, mood**			

BD/BDI	Severity of depressive symptoms.	5-10 min	Emotional functioning and mood
BI	Functional independence in basic activities of daily living (ADLs) such as feeding, bathing, dressing, and mobility.	5-10 min	Physical functioning and self-care
BNT/ BGRT	Confrontational word retrieval and naming ability using picture stimuli.	20-30 min	Language
BSI	Psychological distress across nine symptom dimensions (e.g., depression, anxiety, somatization).	10-15 min	Emotional and psychological functioning
CBT	Not a test but a structured psychotherapeutic approach targeting maladaptive thoughts and behaviors	Typically 12–20 sessions over several months.	Emotional regulation, cognitive restructuring, and behavioral adaptation
CES-D	Screening for depressive symptoms such as low mood, guilt, helplessness, psychomotor retardation, appetite changes, and sleep disturbance	5–10 minutes (CES-D-10) 15–20 minutes (CES-D-20)	Emotional functioning and psychological distress
HADS	Screening for anxiety and depression symptoms in medically ill and general adult populations	5-10 min	Emotional functioning and psychological distress
FEEST	Emotion recognition from facial expressions using prototype and morphed stimuli	10-15 min	Social Cognition and Emotional Processing
MFD	12 dimensions of psychological functioning (e.g., affect regulation, self-observation, reality testing)	Varies	Personality structure and adaptive functioning
RIES	Psychological resilience via self-confidence and self-efficacy	5 min	Emotional Reliance and coping capacity
RLCQ	Stressful life events and their impact using Life Change Units (LCUs)	10-15 min	Psychosocial stress and life event burden
**Global measurements of outcome, health, quality of life**			
EQ-5D	Health-related quality of life across five domains: mobility, self-care, usual activities, pain/discomfort, and anxiety/depression	5 min	Global health status and quality of life
GOS-E	Global functional outcome and recovery after brain injury	15 min	Global functional recovery

GOS	Global functional outcome and recovery after brain injury	10-15 min	Global functional recovery
LiSAT-11	Perceived satisfaction across life domains including relationships, leisure, self-care, and vocational situation	5 min	Quality of life and psychosocial well-being
MFI	Five dimensions of fatigue: general, physical, mental, reduced activity, and reduced motivation	5-10 min	Physical and mental fatigue; quality of life
MRS	Degree of disability or dependence in daily activities post-stroke	5-15 min	Functional independence and global outcome
SF-36	Health-related quality of life across physical, emotional, and social domains; includes physical and mental health composite scores	10-15 min	Global health status with emphasis on physical functioning, mental health, and social well-being
SIP	Perceived impact of illness on physical, emotional, and social functioning; sensitive to changes in health status over time	20–30 minutes (SIP-136) 15–20 minutes (SIP-68)	Global health status with emphasis on physical, psychosocial, and behavioral functioning
SWLS	Global cognitive judgment of life satisfaction	1 min	Subjective well-being and emotional functioning
**Others; Multi-domain**			
NART	Premorbid intelligence using pronunciation of irregular words	5-10 min	Crystallized intelligence (verbal IQ proxy)
RLAS-R/ LCS	Cognitive and behavioral recovery stages following traumatic brain injury	N/A	Cognitive functioning and behavioral responsiveness across 10 recovery levels
RLS85	Level of consciousness and responsiveness in patients with acute brain disorders	1-2 min	Arousal and neurological status (coma scale)
TT	Auditory comprehension and receptive language through verbal commands involving tokens	10-20 min	Language
VTS	Broad cognitive domains including attention, memory, executive function, and psychomotor skills via computerized testing	Varies by Battery	Multi-domain cognitive and psychological assessment
WAB AQ & CQ	Language function (AQ) and broader cognitive functioning (CQ) in individuals with aphasia	30-60 min	Language, auditory comprehension, repetition,

			naming, reading, writing, and nonverbal skills
WASI-II	Verbal, nonverbal, and general cognitive ability through four subtests (Vocabulary, Similarities, Block Design, Matrix Reasoning)	15 minutes (2-subtest form)<br>30 minutes (4-subtest form)	Intelligence (verbal comprehension, perceptual reasoning, and full-scale IQ)

Supplemental Text:

Discussion on MOCA Variants:

MoCA variants exist to accommodate various clinical situations, such as telephone assessment and assessment in patients with severely impaired vision. The T-MoCA is an abbreviated version of the MoCA specifically redesigned and validated for cognitive assessment over the telephone. It tests attention, memory, language, abstraction, and orientation. The MoCA-Blind was redesigned for individuals with visual or motor impairments. It contains questions that test all the domains in the T-MoCA, along with conceptual thinking and calculations. These additional domains are related to executive function, a domain that is frequently impaired in SAH survivors. [135] Since the MoCA-Blind retains more of the full MoCA's domains, it has been found to have superior sensitivity in detecting early and mild cognitive impairment as compared to both the T-MoCA and the MMSE. [157] Its ability to be administered quickly in person or remotely allows for greater participant flexibility and lower attrition. Supplemental Table 5 summarizes the MoCA variants along with strengths and weaknesses.

Supplemental Table S6: MoCA Variants

Variation	Format (with time)	Best for	Key strengths	Key limitations	Why choose for SAH
**Standard MoCA (paper)** [158,159]	30-point, in-person; **~10–15 min**	General clinics and rehab	Broad domain coverage; good sensitivity for mild deficits	Education adjustment needed; practice effects	Executive and attention deficits common post-SAH; baseline and serial tracking

**MoCA-Blind** [78,160]	Paper, no visuospatial items; **~10 min**	Visual impairment, hemianopia	Enables screening when vision limits testing	Reduced coverage of visuospatial/executive functions	SAH with visual pathway injury or severe ocular deficits
**MoCA-Basic** [161]	Simplified language/education demands; **~10–15 min**	Low literacy or limited education	More equitable performance across education levels	Maybe less challenging for higher-functioning patients	Reduced education bias- less dependent on literacy
**Short-Form MoCA (SF-MoCA)** [162]	Abbreviated item set; **~5 min**	Time-limited or high-throughput settings	Faster administration; reasonable accuracy for time-limited settings	Varies by version; lower granularity; validation varies	Quick screen in ICU or early post-bleed when fatigue limits testing
**MoCA 5-Minute protocol (telephone)** [163]	Audio-only adaptation; **~5 min**	Telehealth or remote follow-up	Practical when in-person isn't feasible	Omits visuospatial tasks; risk of under-detection	Longitudinal check-ins during recovery or barriers to travel
**eMoCA/digital MoCA** [164]	Tablet/computer-based; **~10–15 min**	Standardized administration	Automated scoring; version control; remote options	No evidence of being used in SAH; however, is "equivalent" to paper MOCA	Consistency across serial assessments; integrated data capture
**Alternate versions (7.1/7.2/7.3)** [158]	Paper, parallel forms; **~10–15 min**	Serial testing	Minimizes learning effects	No evidence of being used in SAH; however, alternate forms is common in longitudinal recovery testing	Repeated assessments during SAH rehabilitation and return-to-work

Supplemental Table S7: List of Non-standard Acronyms and Abbreviations:

**Abbreviation**	**Definition**
ACE-R	Addenbrooke’s Cognitive Examination - Revised
AMPK	Adenosine Monophosphate-Activated Protein Kinase
aSAH	aneurysmal Subarachnoid Hemorrhage
BGRT	Benton Geometric Recognition Test
BI	Barthel Index
B-MoCA	Blind Montreal Cognitive Assessment
BNIS	Barrow Neurological Institute Screen for Higher Cerebral Functions
BNT	Boston Naming Test
BSI	Brief Symptom Inventory
BVR	Benton Visual Retention
BVRT	Benton Visual Retention Test
CALCAP	California Computerized Assessment Package
CBT	Category-Based Test
CDT	Clock Drawing Test
CFQ	Cognitive Failures Questionnaire
CLOX	Executive Clock Drawing Test
CMI / CMII	Cornell Medical Index
COWAT	Controlled Oral Word Association Test
CSF	Cerebrospinal Fluid

CVLT	California Verbal Learning Test
DCI	Delayed Cerebral Ischemia
DS	Digit Span
DSB	Digit Span Backward
DSF	Digit Span Forward
EQ-5D / EQ5D	EuroQol Five Dimensions Questionnaire
ESS	Epworth Sleepiness Scale
EuroQol	A network of international multidisciplinary researchers devoted to the measurement of health status
FEDA	Fragebogen zur Erfassung der Aufmerksamkeit (Questionnaire for Attentional Functioning)
FEEST	Facial Expressions of Emotion: Stimuli and Tests
FRT	Facial Recognition Test
GCS-E	Glasgow Coma Scale - Extended
German Bourdon-Wiersma	Bourdon-Wiersma Dot Test (German Version)
GPT	Grooved Pegboard Test
H&H	Hunt and Hess Score
HADS	Hospital Anxiety and Depression Scale
HOCOT	Hooper Visual Organization Test
IADL	Instruments of Daily Living
ICH	Intracerebral Hemorrhage
ICP	Intracranial Pressure
ICSD-3	International Classification of sleep disorders 3rd edition
ICU	Intensive Care Unit
IL	Interleukin
JLO	Judgment of Line Orientation
MeSh	Medical Subheadings
MFD	Memory for Designs
MFI	Multidimensional Fatigue Inventory
MMSE	Mini-Mental State Examination

MoCA	Montreal Cognitive Assessment
MRS	Modified Rankin Scale
NART	National Adult Reading Test
naSAH	non-aneurysmal Subarachnoid Hemorrhage
NF-kB	nuclear factor-kappa B
OMCT	Orientation Memory Concentration Test
OSA	Obstructive Sleep Apnea
PRISMA-ScR	Preferred Reporting Items for Systematic Reviews Protocol
PSG	Polysomnography
PSQI	Pittsburgh Sleep Quality Index
RAVLT	Rey Auditory Verbal Learning Test
RCF / RCF-R	Rey Complex Figure / Rey Complex Figure Recall
RCFT	Rey Complex Figure Test
RCSQ	Richards-Campbell Sleep Questionnaire
RLAS-R	Rancho Los Amigos Scale - Revised
ROCF Copy	Rey-Osterrieth Complex Figure Copy
ROCFT	Rey-Osterrieth Complex Figure Test
RWT	Regensburger Wortflüssigkeitstest (Word Fluency Test, German)
SAH	Subarachnoid Hemorrhage
SBT	Short Blessed Test
SCI	Sleep Condition Indicator
SCWT	Stroop Color-Word Test
SF-36	Short Form-36 Health Survey
SRB:1	Sentence Repetition Battery 1
SRT	Selective Reminding Test
STOP-BANG	Snoring, Tiredness, Observed apneas, Pressure (high blood pressure), Body Mass Index (BMI), Age, Neck circumference, and Gender
SWLS	Satisfaction With Life Scale

SWR	Stroop Word Reading
TBI	Traumatic Brain Injury
TBK	TRAF-associated NF-κB Activator -Binding Kinase
Th17	T helper 17 cell
TICS	Telephone Interview for Cognitive Status
T-MoCA	telephone MoCA
TMT-A	Trail Making Test Part A
TMT-B	Trail Making Test Part B
TNF	Tumor Necrosis Factor
Treg	Regulatory T cell
TT	Token Test
VAMPT	Verbal Adult Memory Processes Test
VPA / vPAL	Verbal Paired Associates / Verbal Paired Associate Learning
VRT	Vienna Reaction Test
VSAT	Visual Selective Attention Test
VTS	Vienna Test System
WAB AQ & CQ	Western Aphasia Battery - Aphasia Quotient & Cortical Quotient
WAIS-R	Wechsler Adult Intelligence Scale - Revised
WASI-II	Wechsler Abbreviated Scale of Intelligence, Second Edition
WCST	Wisconsin Card Sorting Test
WMS-R	Wechsler Memory Scale - Revised
